# Spatial phylogenetics of the native California flora

**DOI:** 10.1186/s12915-017-0435-x

**Published:** 2017-10-26

**Authors:** Andrew H. Thornhill, Bruce G. Baldwin, William A. Freyman, Sonia Nosratinia, Matthew M. Kling, Naia Morueta-Holme, Thomas P. Madsen, David D. Ackerly, Brent D. Mishler

**Affiliations:** University and Jepson Herbaria and Department of Integrative Biology, University of California, 1001 Valley Life Sciences Building, Berkeley, CA 94720 USA

**Keywords:** California flora, Spatial phylogenetics, Phylogenetic diversity, Phylogenetic endemism, CANAPE, Evolution, Species distribution modeling

## Abstract

**Background:**

California is a world floristic biodiversity hotspot where the terms neo- and paleo-endemism were first applied. Using spatial phylogenetics, it is now possible to evaluate biodiversity from an evolutionary standpoint, including discovering significant areas of neo- and paleo-endemism, by combining spatial information from museum collections and DNA-based phylogenies. Here we used a distributional dataset of 1.39 million herbarium specimens, a phylogeny of 1083 operational taxonomic units (OTUs) and 9 genes, and a spatial randomization test to identify regions of significant phylogenetic diversity, relative phylogenetic diversity, and phylogenetic endemism (PE), as well as to conduct a categorical analysis of neo- and paleo-endemism (CANAPE).

**Results:**

We found (1) extensive phylogenetic clustering in the South Coast Ranges, southern Great Valley, and deserts of California; (2) significant concentrations of short branches in the Mojave and Great Basin Deserts and the South Coast Ranges and long branches in the northern Great Valley, Sierra Nevada foothills, and the northwestern and southwestern parts of the state; (3) significant concentrations of paleo-endemism in Northwestern California, the northern Great Valley, and western Sonoran Desert, and neo-endemism in the White-Inyo Range, northern Mojave Desert, and southern Channel Islands. Multiple analyses were run to observe the effects on significance patterns of using different phylogenetic tree topologies (uncalibrated trees versus time-calibrated ultrametric trees) and using different representations of OTU ranges (herbarium specimen locations versus species distribution models).

**Conclusions:**

These analyses showed that examining the geographic distributions of branch lengths in a statistical framework adds a new dimension to California floristics that, in comparison with climatic data, helps to illuminate causes of endemism. In particular, the concentration of significant PE in more arid regions of California extends previous ideas about aridity as an evolutionary stimulus. The patterns seen are largely robust to phylogenetic uncertainty and time calibration but are sensitive to the use of occurrence data versus modeled ranges, indicating that special attention toward improving geographic distributional data should be top priority in the future for advancing understanding of spatial patterns of biodiversity.

**Electronic supplementary material:**

The online version of this article (doi:10.1186/s12915-017-0435-x) contains supplementary material, which is available to authorized users.

## Background

Identifying areas with highly concentrated richness or endemism has long been the desired objective of biodiversity assessments. Such studies have most commonly focused on species alone, an approach that can provide valuable insights but gives an incomplete picture of diversity since it fails to incorporate their evolutionary relationships among species. Extensive digitization of museum specimens and rapid accumulation of DNA sequence data mean that phylogenetic approaches can now be used to address questions of the origins and maintenance of diversity and endemism at a macroecological scale [1–4]. While it once took years of field work and experience to gather enough knowledge to subjectively judge richness and endemism, see, e.g., Stebbins and Major’s [5] classic study of Californian plants, this can now be done systematically and reproducibly using spatial data for whole floras (collated over centuries of field work) in combination with DNA phylogenies to discover significant evolutionarily and ecologically distinct concentrations of biodiversity.

### Measuring endemism

It is important to understand that there are two different concepts of endemism. *Absolute endemism* refers to the complete restriction of a taxon to a particular area; for example, Monterey cypress (*Hesperocyparis macrocarpa*) is natively restricted to a small portion of Monterey County in California. A taxon can also be endemic to a larger area; for example, the genus *Sequoia* is endemic to the California Floristic Province (CA-FP). The concept of *relative endemism* differs by considering endemism on a continuous scale, as the inverse of the range size of a taxon ranging from 1 (= absolute endemism) to 0 (= occurring everywhere). The region that is investigated can be of any size, and is partitioned, usually into grid cells, so that the endemism values can be judged for each partition of the region. Thus, there is another important distinction to be made, between endemism as a property of a taxon versus a property of the flora of a region — a distinction that is orthogonal to the absolute-relative distinction. The weighted endemism (WE) score for a taxon is the inverse of its range size (e.g., the number of grid cells in which it occurs); the WE score for a region is the sum of the WE scores for each taxon in that region [6]. In this paper we exclusively use “endemism” in the relative sense, and apply the term exclusively to geographic regions.

### Phylogenetic diversity and endemism

Phylogenetic diversity (PD) was described by Faith [7] as the sum of the phylogenetic branch lengths present in a given area, including all branches that connect the taxa present to the base of the tree. Considering the application of PD to range-restricted organisms, Faith [7] and Rosauer et al. [8] both described approaches to define and quantify phylogenetic endemism (PE). Faith’s approach determined the parts of a phylogenetic tree that are completely restricted to a given region, an approach that could be called “absolute PE.” Alternatively, applying the concept of WE described by Crisp et al. [6] to phylogenetics, Rosauer et al. [8] developed an approach that could be called “weighted PE.” They range-weighted the branches of a phylogeny by dividing each branch (terminal and deeper) by its range size (where the range of deeper branches is taken to be the union of the ranges of its descendants) and giving each grid cell a PE value based on the sum of the range-weighted branch lengths included. This measure, which we use in this paper, highlights regions occupied by taxa with relatively long branches, small geographic ranges, and especially the combination of the two.

Mishler et al. [1] further developed the concepts of PD and PE, adding derived metrics called relative phylogenetic diversity (RPD) and relative phylogenetic endemism (RPE) that compare PD or PE measured on the original tree with PD or PE measured on a comparison tree having the same topology but with all branch lengths equal. These metrics were designed to examine the distribution of branch lengths in an area. Mishler et al. [1] also developed a randomization test for PE and RPE, called categorical analysis of neo- and paleo-endemism (CANAPE). This test is able to identify significant concentrations of neo-endemism (i.e., range-restricted short branches) or paleo-endemism (i.e., range-restricted long branches) as well as mixtures of the two, and was the first to allow quantitative mapping of concentrations of these endemism types across a landscape. In this way, phylogenetic approaches add an evolutionary dimension to understanding spatial patterns of diversity and endemism [1–4, 9, 10]. In addition to simply identifying areas of taxon richness and endemism, incorporating the phylogeny facilitates investigations of the potential evolutionary and ecological causes (e.g., centers of divergence or competitive exclusion) of significant concentrations of diversity and endemism.

### The effect of dating phylogenies and missing spatial data

All CANAPE studies to date have used inferred genetic branch lengths (i.e., phylograms), and therefore interpretations of neo- and paleo-endemism have referred to areas that contain a high concentration of range-restricted short or long branches in terms of amount of genetic change [1–4]. It has not been determined to date what happens to the interpretations when a time-constrained phylogeny (i.e., a chronogram) is used for the branch lengths in CANAPE.

The use of point data from herbarium specimens in spatial studies of diversity is also not without contention. Although available specimen datasets are becoming huge, thanks to ongoing digitization activities, they still fail to represent the full distribution of all taxa. By underestimating the complete ranges of taxa, endemism and richness patterns may be affected. Baldwin et al. [11] discussed issues of sampling with herbarium data in detail, and argued that typical collector’s goals likely result in specimen data that are biased towards both diversity and endemism. Nonetheless, it is clear that taxon ranges are underestimated to some extent with herbarium data, and areas that have lower sampling will not reflect the full set of taxa that occur there. One approach to overcome this problem is to aggregate collections into spatially larger grid cells, but this method results in a coarser pattern that is hard to interpret. An alternate approach is to use predicted ranges from species distribution modeling (see, e.g., Loarie et al. [12]). Species distribution modeling of taxon ranges is useful for exploring the potential area in which an organism could occur, either now or into the future [2] and could be used to fill in missing distributional data. The true range size of a taxon presumably lies somewhere between that documented by occurrence records and the distribution of suitable environments.

### California

The California flora is relatively well known and is one of the most diverse and threatened in the temperate world, with more than 25% of species restricted solely to the state [13], exceeding the number of native and endemic vascular plant taxa in any other state of the USA [14, 15]. California also forms the majority of the CA-FP [16, 17], one of only two North American areas among the 36 recognized global biodiversity hotspots [18]. Much of California occurs in an isolated Mediterranean-like climatic area [19], with the closest climatically similar environment occurring in Chile, South America. The climatic isolation coupled with a complex geological history, a diversity of elevational environments, and a rich mosaic of soil types have all been proposed as significant to the formation of the unique flora [17, 20]. Thus, California is an outstanding model system in which to examine the application of existing spatial phylogenetic methods and to explore some extensions of them.

Curiosity about the origins of California plant diversity and endemism has led to studies investigating the influence of ecological and biogeographic factors, many of which have been at the forefront of plant evolutionary research [21–28]. Of all Californian studies, arguably the most influential was the statewide research of Stebbins and Major [5], who popularized the terms neo- and paleo-endemism in reference to range-restricted taxa that are either newly evolved (e.g., some Asteraceae or Poaceae) or the last living example of a once more dominant group (e.g., *Sequoia*). Using species from 70 large to medium-sized genera and 178 “relict” genera (based on the assumption that their nearest relatives were extinct or widely disjunct), they identified three coastal subdivisions with an over-abundance of endemics and two regions in Northwestern and far southern California with high proportions of paleo-endemics.

Thirteen years later, Raven and Axelrod [17] expanded Stebbins and Major’s work to the entire California vascular flora, discussing the role that soil type played in creating endemism and addressing modes of evolution, with detailed accounts of diversity in several major genera or families that have a large Californian presence. As both of these works predated modern molecular phylogenetics, application of the neo- and paleo-endemic categories was based on taxonomic ranks and expert judgments of what appeared to be taxonomically rich groups having presumably undergone recent speciation, versus those with few known close relatives. More recent analyses of Californian floristic diversity or endemism have been attempted at finer geographic and ecological scales, and multiple conclusions have been drawn, including the primacy of climatic factors, especially mean precipitation, as the strongest predictors of high diversity [29], or elevational or habitat heterogeneity [30] as the most important determinant of plant diversity. Thorne et al. [31] identified the Sierra Nevada as a diversity hotspot, and the Central Coast Ranges and Transverse Ranges as endemism hotspots, with the greatest concentrations of range-restricted endemics found in the Channel Islands; they concluded that topographic complexity, soil diversity, and geographic isolation were likely most important in explaining plant richness and endemism in California.

A phylogenetic examination of neo-endemism, using 15% of California’s endemic species and 800 non-overlapping areas, was conducted by Kraft et al. [32]. They found that the central coast, Sierra Nevada, and part of the Transverse Ranges had the highest concentrations of neo-endemics within the state. Kraft et al. [32] also showed that the deserts, including the Great Basin, had the youngest neo-endemics, and importantly, that a large proportion of Californian areas with high neo-endemism were outside of protected lands. More generally, from reviewing published phylogenetic data for Californian vascular plants, Baldwin [15] concluded that most lineages with endemic species diversity in the CA-FP — and especially those that are primarily herbaceous — can be regarded as neo-endemic, with diversification since the mid-Miocene onset of transition to a summer-dry climate and with closest relatives elsewhere in North America, usually in the west or southwest.

A recent paper by our group [11] examined patterns of species richness and endemism in the California flora, and found that some areas of high species richness also show significantly high endemism (i.e., the WE of the region as defined above), but such an association was not generally found. For example, in Southwestern California, species richness is high in much of the Peninsular and Transverse Ranges, but significantly high endemism is primarily confined to the San Bernardino and Santa Rosa Mountains. Conversely, species richness is low and endemism is significantly high in the Channel Islands and in major parts of the Death Valley region.

Here, we utilized the full Californian vascular plant spatial dataset of Baldwin et al. [11] and added a phylogenetic element. We used both newly generated DNA sequences and sequences obtained from GenBank to estimate the most comprehensive phylogeny of all Californian vascular plant lineages ever assembled, and to investigate the distribution of PD and endemism across the Californian landscape in relation to climatic variables. We addressed several novel methodological and biological questions, using California as our study system. (1) How do patterns seen using phylogenetic methods differ from those seen in a previous study of California that used traditional taxon-based measures? (2) How are PD and endemism patterns affected when using a dated chronogram rather than a phylogram? (3) How are PD and endemism patterns affected when using modeled ranges versus herbarium records? (4) How are diversity and endemism patterns affected by phylogenetic uncertainty? (5) Can correlations be found between environmental variables and significant concentrations of PD and endemism?

## Results

### Tree topology

The 10 maximum likelihood tree searches resulted in phylogenies with similar but not identical topologies, as measured by Robinson-Foulds distances (Additional file 1: Table S1). Downstream spatial phylogenetic analyses were performed on all 10 trees to test for the effects of phylogenetic uncertainty; however, we selected a single tree to use for most analyses based on the criterion that it had the highest log-likelihood value of those trees whose topology reflected the ordinal and family membership as outlined in Angiosperm Phylogeny Group III (APG III) [33]. Out of the 10 maximum likelihood trees, the selected tree for analyses had the second highest log-likelihood value overall (Additional file 1: Table S1). This tree traced with APG III orders is shown in Additional file 2: Figure S1. Bootstrap support values for this tree can be viewed on Additional file 3: Figure S2.

### Spatial phylogenetic results for California using the phylogram and herbarium records

#### Richness and diversity

**Fig. 1:**
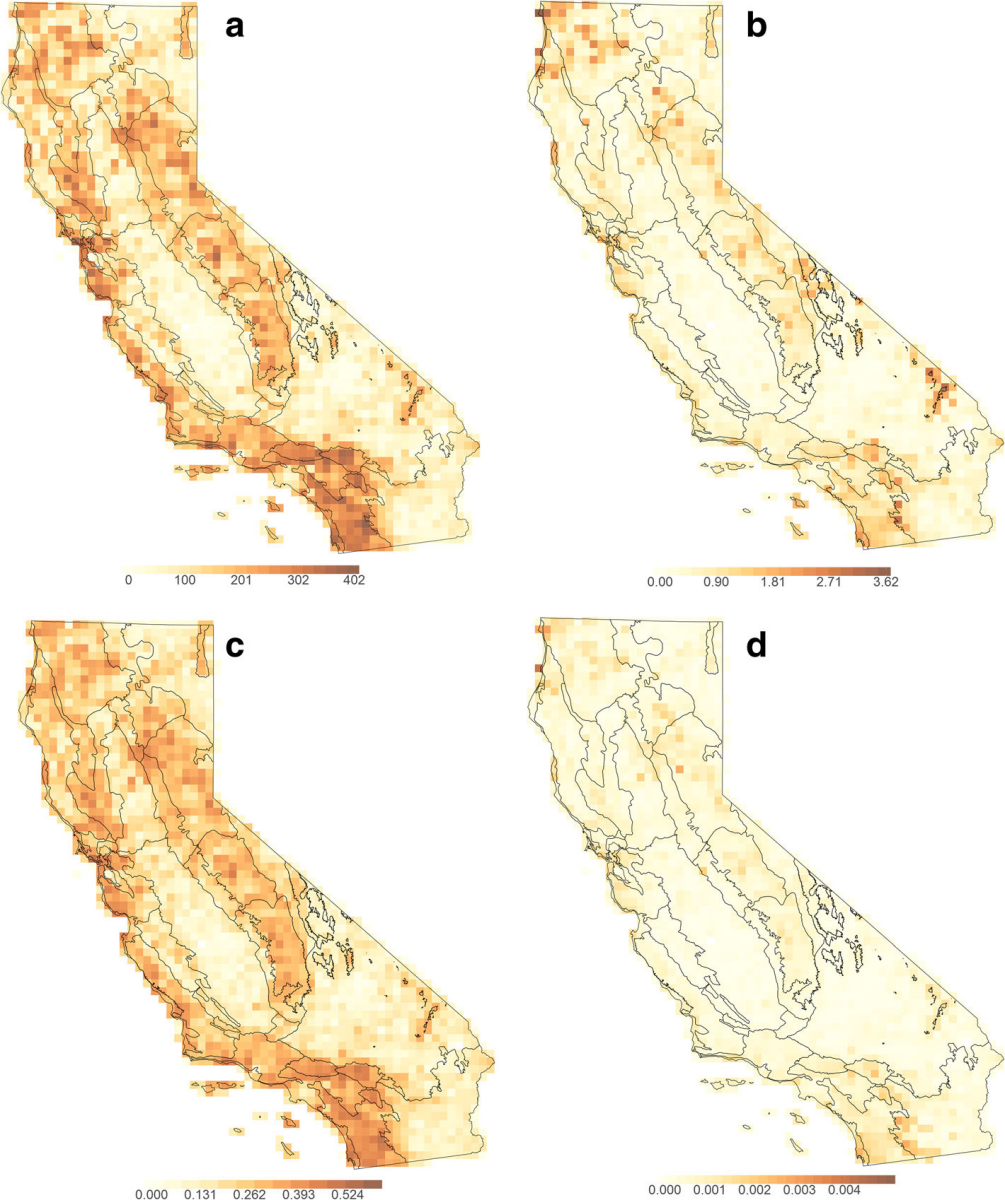
Observed results, mapped with an overlay of the Jepson bioregions of California. **a** Richness of the 1083 OTUs. **b** Weighted endemism of the 1083 OTUs. **c** Phylogenetic diversity, which shows a very similar pattern to richness. **d** Phylogenetic endemism, which shows a similar pattern to weighted endemism with some noticeable differences in the eastern desert mountains, northwest, Bay Area, and southwest

In all analyses using the raw herbarium record locations without niche modeling, the mountainous areas of California showed the highest observed TR and PD (Fig. 1 and Additional file 4: Figure S3). Concentrations of high WE and PE differed, however (Fig. 1). The eastern desert mountains were high for WE but not for PE. The northwest and the southwest also showed relatively high WE but were not particularly high in PE. When analyzed as a subset, the angiosperm richness pattern closely matched that of the all-vascular-plant analysis, showing the major influence that the angiosperms have on overall patterns (Additional file 4: Figure S3). In contrast, when analyzed separately, gymnosperms and pteridophytes each showed high TR only in the mountainous and coastal areas of California (Additional file 4: Figure S3). Similar patterns were observed in analysis of clades restricted to California (Additional file 4: Figure S3).

**Fig. 2:**
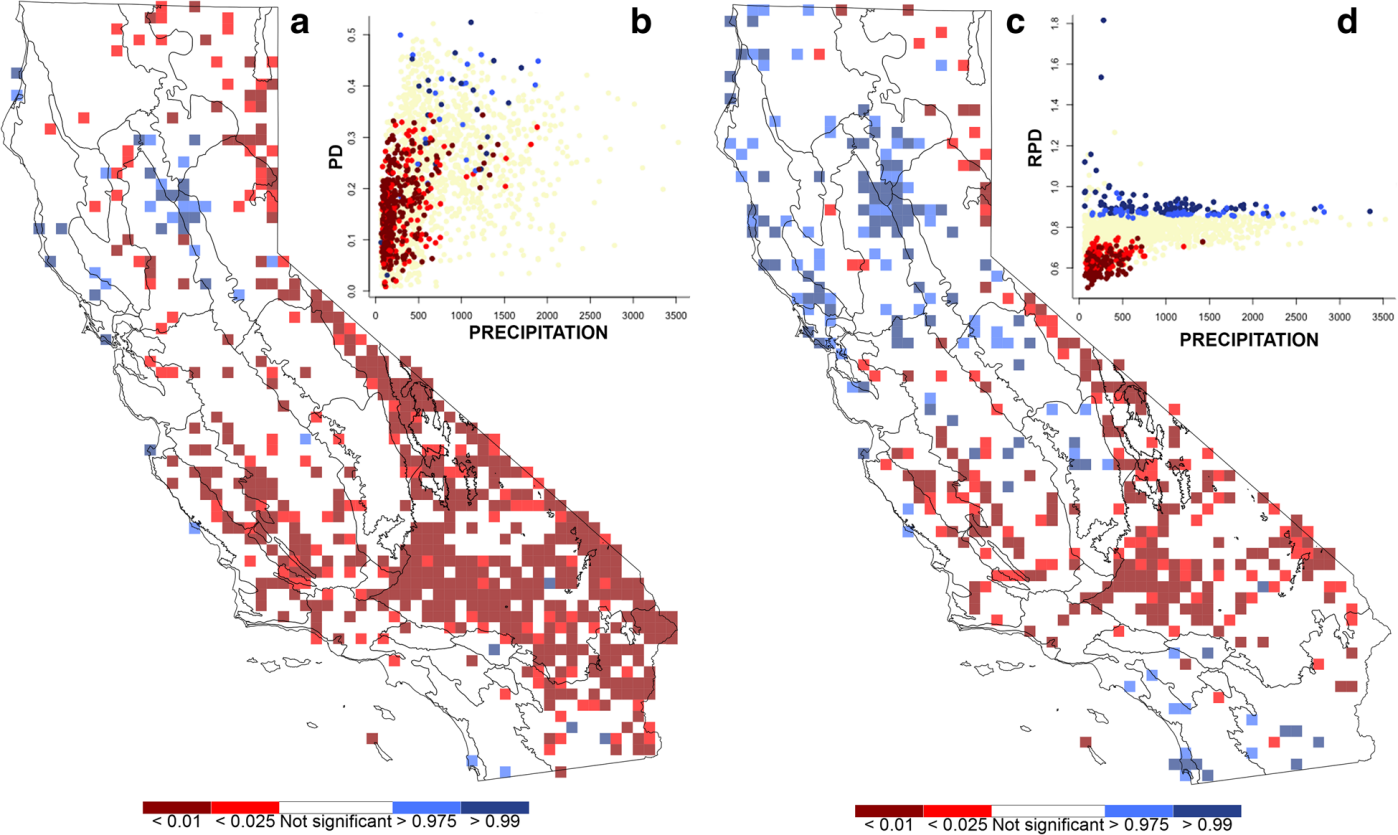
Results of randomization tests, mapped with an overlay of the Jepson bioregions of California shown in the background. **a** Randomized phylogenetic diversity (*PD*). Cells in *red* have a significant under-representation of the phylogeny and are widespread in the southern half of the state. Cells in *blue* have a significant over-representation of the phylogeny and are scattered with a concentration in the northern Great Valley and Sierra foothills. **c** Randomized relative phylogenetic diversity (*RPD*). Cells in *red* have a significant concentration of short branches and occur in the desert and South Coast Ranges. Cells in *blue* have a significant concentration of long branches and occur in the northern Great Valley and Sierra foothills, North Coast Ranges, Bay Area, and southwest. Insets **b** and **d** show the significant cells for the two metrics plotted against precipitation for the area in which they occur. Cells with significantly low PD and RPD are concentrated at the low end of the precipitation gradient

The PD randomization for the full vascular flora indicates that there is extensive phylogenetic clustering (i.e., significantly low PD) in the South Coast Ranges, southern Great Valley, and deserts of California (Fig. 2a). Only one major concentration of phylogenetic over-dispersion (i.e., significantly high PD) was apparent, in the northern Great Valley and adjacent Sierra Nevada foothills. The RPD randomization indicated significant concentrations of short branches (i.e., significantly low RPD) in the Mojave and Great Basin Deserts and the South Coast Ranges (Fig. 2c). There were significant concentrations of long branches (i.e., significantly high RPD) in the northern Great Valley and northern Sierra Nevada and in the northwestern and southwestern parts of the state.

**Fig. 3:**
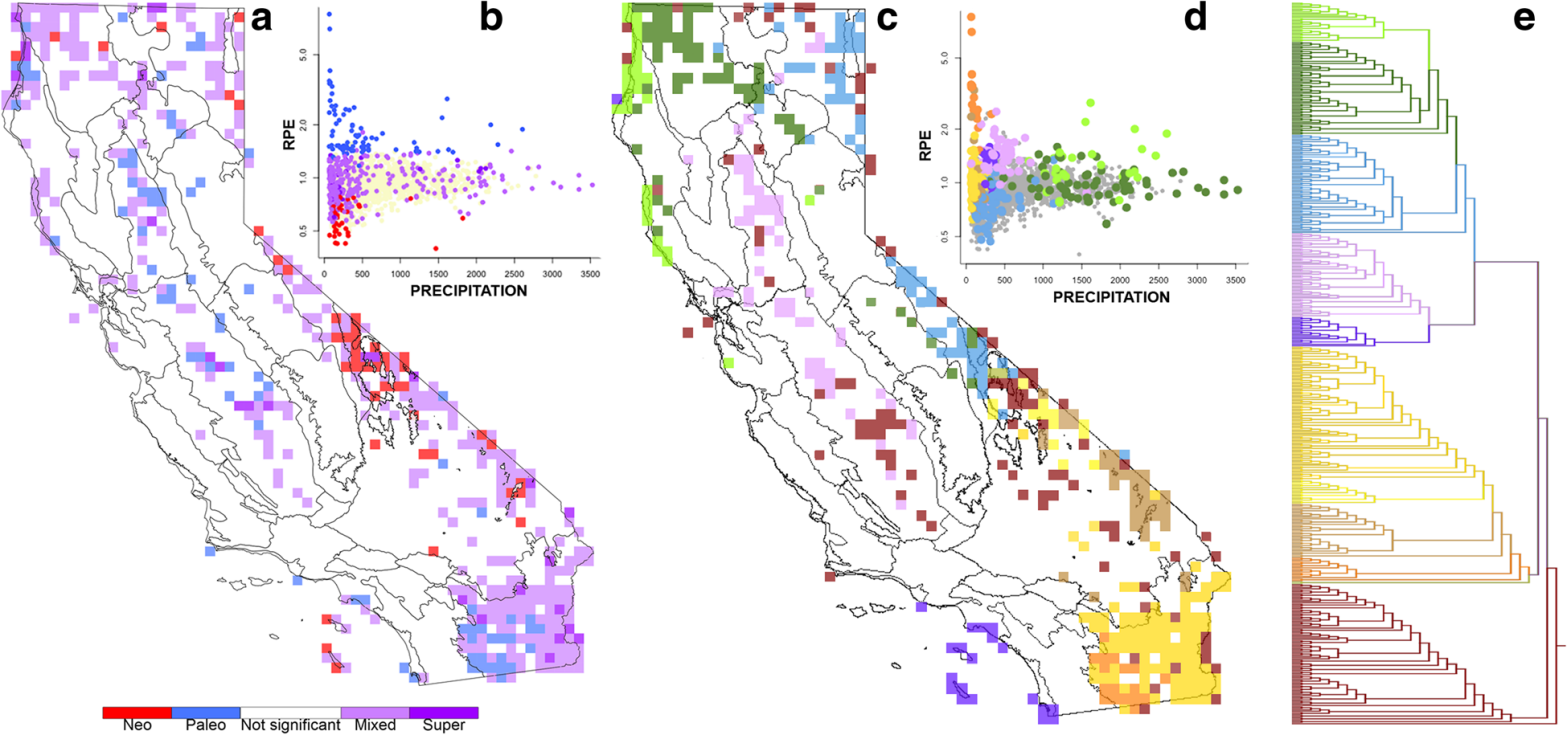
Results of CANAPE analysis of all vascular plants, mapped with an overlay of the Jepson bioregions of California. **a** 15 × 15 km grid cells colored that showed significantly high phylogenetic endemism (PE) in CANAPE; *red* shows centers of neo-endemism, *blue* shows centers of paleo-endemism, and *two shades of purple* show two levels of mixed endemism. **b** A scatterplot showing annual precipitation versus relative phylogenetic endemism (*RPE*), colored to match the cells in **a**. **c** A range-weighted phylogenetic turnover analysis among cells that showed significant phylogenetic endemism in CANAPE. The colors indicate the corresponding clusters in **e** and show five major groupings. The primarily northwest cells (*dark green*) and northern coast cells (*light green*) together form a distinct cluster most similar to the Great Basin Desert cluster (*light blue*), while the northern Great Valley cells cluster (*pink*) with the Channel Island and south coast cells (*purple*). The desert cells form five distinct clusters (*yellows, orange, browns*). Many geographically dispersed cells did not form a distinct cluster (*maroon*). **d** A scatterplot showing annual precipitation versus RPE, colored to match the corresponding clusters in an Unweighted Pair Group Method with Arithmetic Mean (UPGMA) cluster analysis based on range-weighted phylogenetic turnover, colored to match the corresponding cells in **c** and **d**; cells that cluster together closely share similar branches in the phylogeny. Significantly high PE is concentrated in low precipitation environments, except for the northwest, probably indicating different processes affecting endemism in different lineages and in different locations

Many locations showed significant phylogenetic endemism in CANAPE with the full dataset (Fig. 3) and angiosperms alone (Additional file 4: Figure S3). Northwestern California, the Great Valley, and the western Sonoran Desert showed significant concentrations of paleo-endemism. The White-Inyo Range, northern and eastern Mojave Desert, and southern Channel Islands (Santa Catalina and San Clemente Islands) showed significant concentrations of neo-endemism. The Klamath Ranges, North Coast, northern North Coast Ranges, Farallon Islands, San Joaquin Valley, Sonoran Desert, Modoc Plateau, Great Basin Desert, Death Valley region, and the eastern and southern Mojave Desert, including the eastern desert mountains, showed mixed endemism. Analyzed alone, gymnosperms and pteridophytes (Additional file 4: Figure S3) had significant areas of PE in the northwest (both), Central Coast (gymnosperms), and the extreme southwest corner of the state (pteridophytes). The CANAPE analysis of taxa restricted to California retained extensive mixed endemism in the Klamath Ranges, to the interior, and considerable neo-endemism in the White-Inyo Range and Death Valley region, and increased endemism across all of the Channel Islands, but showed a reduction of significant endemism in the Sonoran Desert.

#### Turnover among cells showing significant PE

The range-weighted dissimilarity analysis of CANAPE cells recovered a number of major, geographically distinct clusters representing regions whose endemic flora shares a unique evolutionary history (Fig. 3c and e; colors mentioned below refer to this figure). Certain cells (shown in dark red) were highly distinct from each other and did not form a geographically continuous cluster with any other cells. Cells from the Mojave and Sonoran deserts (shown in yellow and gold) clustered together in five subgroups that were deeply dissimilar to all other clusters. Cells from the northwest (Klamath, North Coast, and outer North Coast Ranges) and High Cascade Ranges, High Sierra Nevada, and northern White-Inyo Range (shown in green) formed two distinct sister clusters that in turn were similar to a cluster (shown in light blue) of Great Basin Desert cells. Cells from the Great Valley (shown in pink) and the southern Channel Islands (shown in purple) formed distinct clusters that were most similar to each other.

### The effects of using chronograms versus the phylogram

**Fig. 4:**
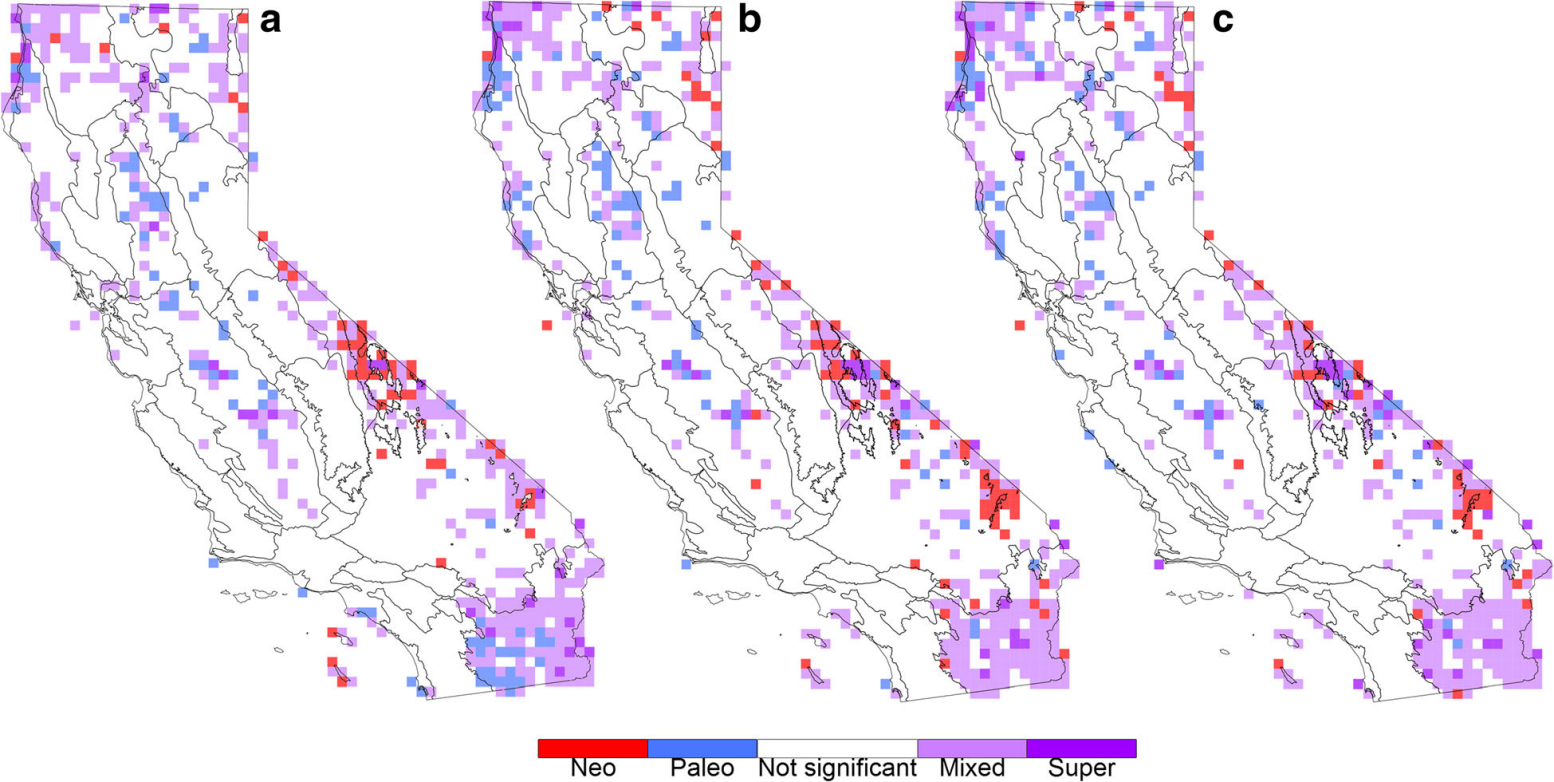
A comparison of significant CANAPE cells for all vascular plants. Using the topology of **a** the original phylogeny, **b** a root-calibrated phylogeny, and **c** a phylogeny dated with 55 calibration points. The location of significant CANAPE cells remains largely stable, but for some cells the interpretation of endemism category changes

The two different time calibration schemes used (root only versus 55 calibrations) had a major effect on the distribution of branch lengths (see Additional file 5: Figure S4 for an overview of all tree shapes used), and while the overall spatial patterns of significance were similar to those using the uncalibrated phylogram, some differences were seen (Additional file 6: Figure S5). The primary difference was that additional cells showing significant PD and RPD appeared in the northwest and the southern Sierra Nevada for both chronograms as compared to the phylogram. The location of cells showing significance in CANAPE also remained relatively constant regardless of whether an uncalibrated, minimally calibrated, or fully calibrated tree was used (Fig. 4). However, the classification of the significant CANAPE cells did change in some cases when using the chronograms. For example, as compared to the vascular plant analysis using the phylogram, in the analysis using the chronograms concentrations of paleo-endemism in the Sonoran Desert became classified as mixed endemism, areas of mixed endemism changed to neo-endemism in the White-Inyo Range, and some areas of mixed endemism changed to paleo-endemism in the North Coast (Fig. 4b and c). Changes in CANAPE interpretation were also noticeable in the subset analyses, especially in the gymnosperm and pteridophyte analyses (Additional file 7: Figure S6).

### Effect of phylogenetic uncertainty

The analyses of CANAPE using the maximum likelihood trees resulting from 10 different analyses showed very little discernable difference in the overall pattern (Additional file 8: Figure S7).

### Spatial phylogenetic results using modeled ranges versus herbarium record localities

**Fig. 5:**
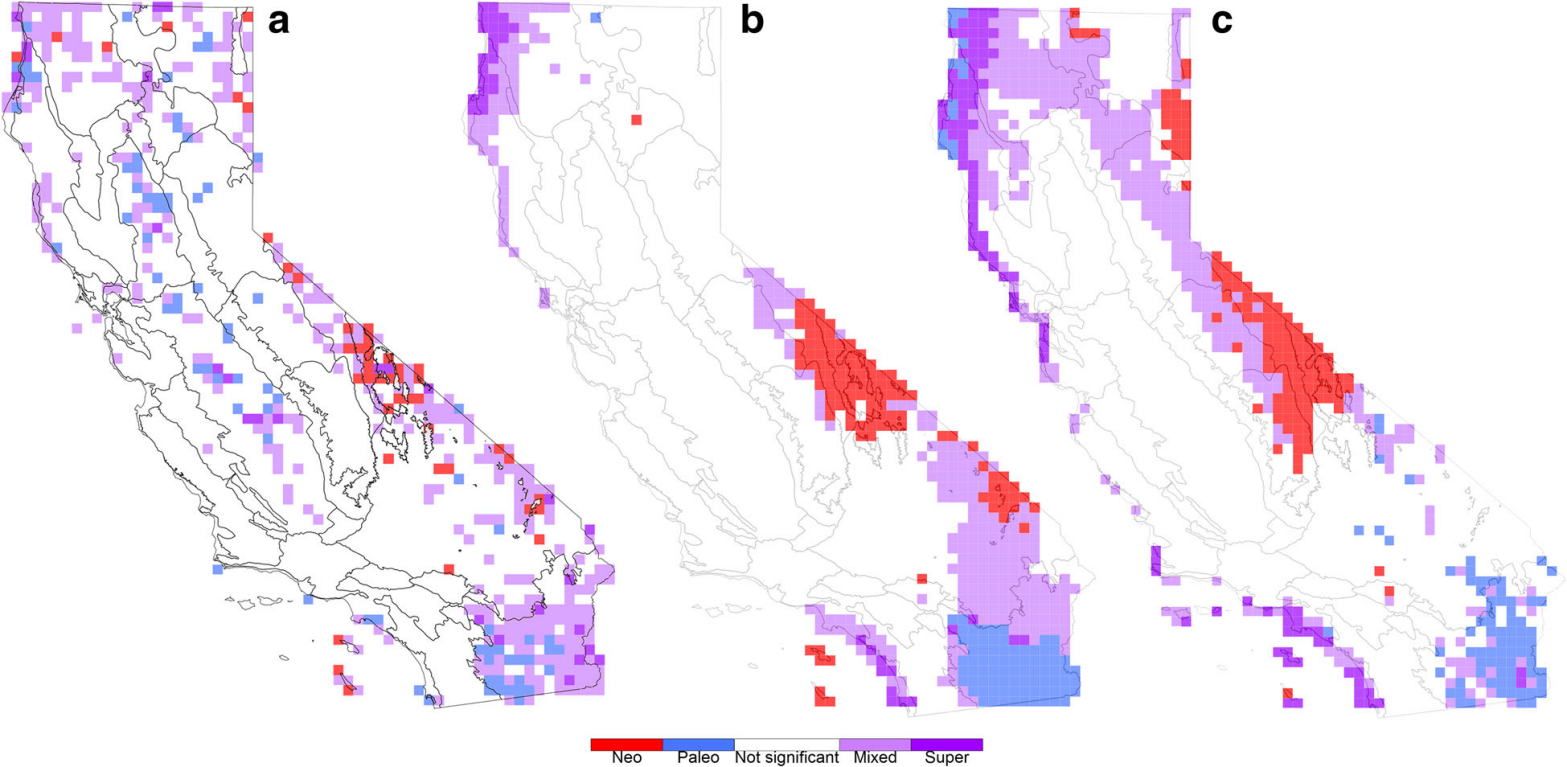
A comparison of significant CANAPE cells for all vascular plants. Resulting maps of analysis using information from **a** the point-occurrence data, **b** a distance hybrid modeling approach, and **c** a binary Maxent modeling approach

**Fig. 6:**
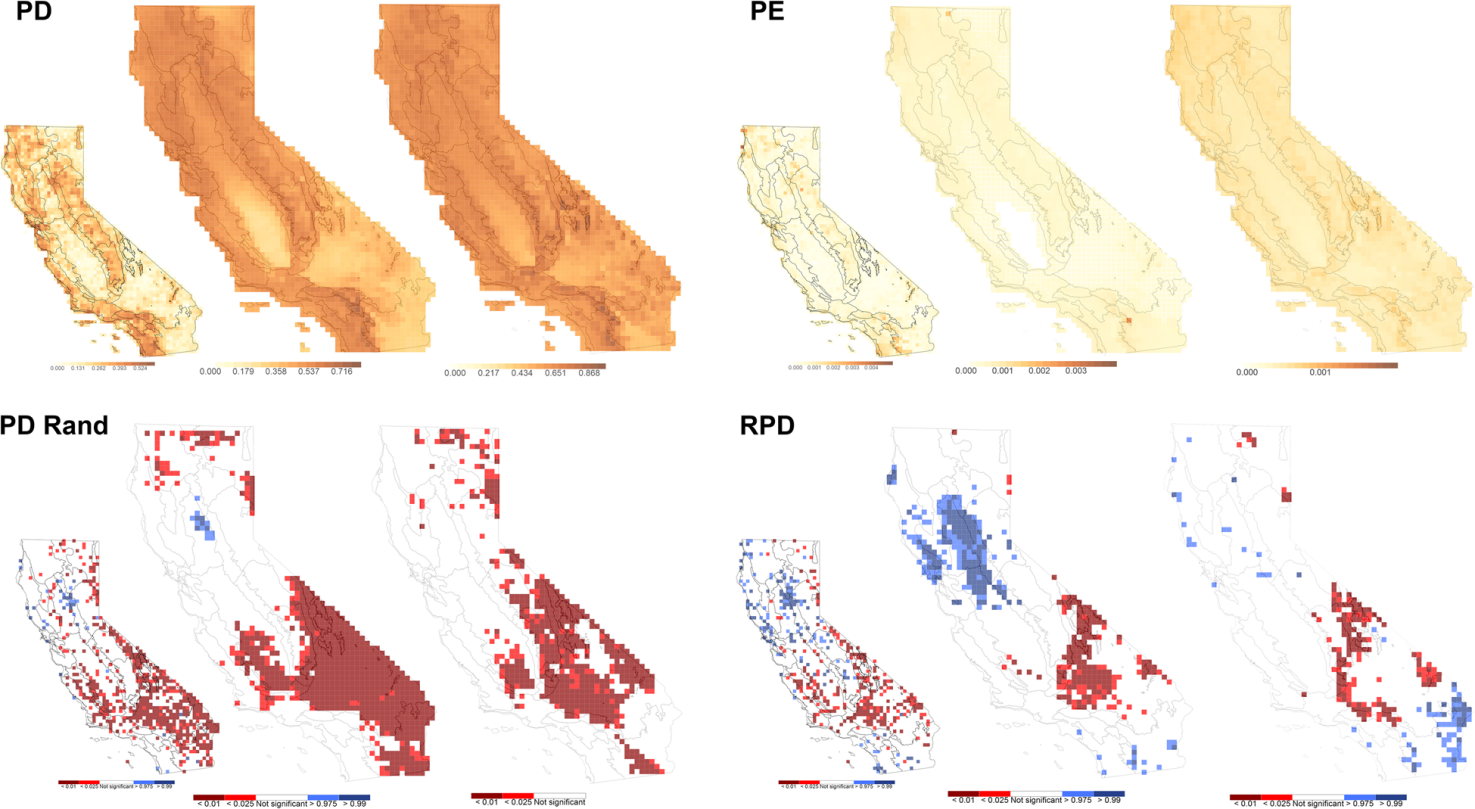
Point-occurrence results compared against range modeled results. A comparison of results as originally measured with the point-occurrence data (*inset left* in each case) to those measured using, in order for each metric, a distance hybrid modeling approach and a binary Maxent modeling approach

**Fig. 7:**
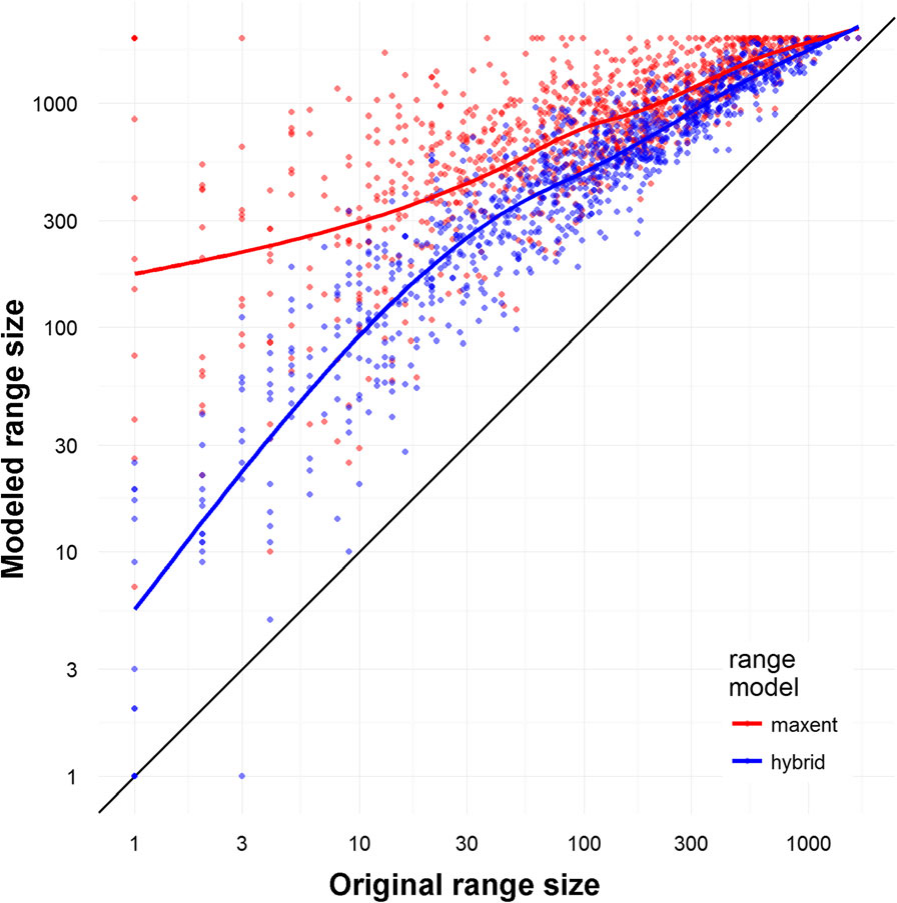
A graphical representation of the range-size expansion seen after modeling. The *X*-axis represents the number of cells in which specimens of an OTU occurred, and the *Y*-axis represents the number of cells in which they were projected to occur after modeling. *Red* represents the binary Maxent modeling, and *blue* represents the distance hybrid approach. The expansion is greater in range-restricted taxa than in widespread taxa

Analyses using modeled ranges yielded considerably different results than those using herbarium occurrence records (Figs. 5 and 6). Unsurprisingly, distribution-model projections for a given OTU generally occupied more grid cells than did the raw specimen collection points, with standard Maxent models predicting larger ranges for taxa than Maxent models with distance constraints (Fig. 7). Compared to the patterns observed using herbarium occurrence records, richness and PD of the mountainous areas of California were greatly amplified and more geographically continuous in both analyses using modeled ranges (Fig. 6 and Additional file 9: Figure S8). However, WE was reduced (because of the larger taxon ranges) compared to results using herbarium occurrences and also was more geographically even. Randomized PD and RPD results from modeled ranges excluded some areas of significance that were identified using occurrence records (Fig. 6 and Additional file 9: Figure S8). In contrast, other concentrations of significant cells were much larger using modeled ranges than occurrence records, for example, for the deserts in PD, northern Great Valley in RPD (for distance hybrid range modeling), and eastern Mojave in RPD (for Maxent range modeling).

CANAPE patterns were also affected by both standard Maxent modeling and the more conservative distance hybrid approach (Fig. 5). Compared to raw point records, both modeling approaches removed significant PE from the Great Valley and greatly increased the size of concentrations of neo-endemism east of the Sierra Nevada and paleo-endemism in the Sonora Desert. With unconstrained Maxent modeling of ranges, CANAPE showed significant mixed endemism along much of the northern California coast and in the Sierra Nevada (Fig. 5c).

### Climate-phylodiversity relationships

Comparing PD, RPD, and RPE with climatic variables (Additional file 10: Figure S9) showed that the low end of the precipitation spectrum contained a disproportionate share of significant cells (Fig. 3b and d). This pattern was particularly striking for RPE, for which drier regions (annual precipitation less than 500 mm) represent 58.2% of all cells (1136/1953) but 73.7% of significant cells (353/479). Broken into three categories, 93% of neo-endemism (40/43), 70% of paleo-endemism (55/78), and 72.1% of mixed endemism (258/358) occur at precipitation less than 500 mm. On the other hand, significant cells were more evenly distributed across gradients of summer and winter mean temperatures as well as water deficit (Additional file 10: Figure S9). Coloring the cells by their cluster as determined by the range-weighted turnover analysis (Fig. 3d) showed that geographic groupings of significant cells in CANAPE had similar climates, especially in precipitation levels.

## Discussion

### Empirical findings

Baldwin et al. [11] determined areas of richness and endemism in California using a species-level geospatial dataset without a phylogenetic component. The present study took the next step of adding a phylogeny, which considers evolutionary differences among co-occurring taxa allowing for community phylogenetics inference [34] at a macroecological scale, as well as the ability to distinguish areas of neo-, paleo-, and mixed endemism [1, 3].

When the OTUs of this study were analyzed without using the phylogeny, the resulting patterns of richness and endemism of these terminal taxa (Additional file 11: Figure S10) were quite similar to those resulting from the species-level analysis. The minor differences between the results of Baldwin et al. [11] and our study were most likely due to the different taxonomic levels treated. For example, the northern Channel Islands had low OTU endemism and richness in the present study but significantly high species endemism in the study of Baldwin et al. [11]. The mountains of California showed the highest observed taxon richness and endemism in our analyses, consistent with the findings of the species-based analysis of Baldwin et al. [11]. Those results more generally conform to results of earlier studies indicating that areas of highest species richness and endemism in California are of high topographic and edaphic heterogeneity, especially in the CA-FP (e.g., [15, 31, 32]). It would be reasonable to hypothesize that the spatial truncation of taxon ranges at the state boundary might influence WE results by underestimating the global range size of non-California-endemic taxa, increasing apparent endemism in areas near the state border. However, Baldwin et al. [11] showed that most of the main centers of significant endemism persisted even when only California-endemic species were analyzed.

Incorporating phylogeny into the analyses recovered new and different patterns from those in the non-phylogenetic analyses of terminal taxa (either species or OTUs), allowing new interpretations of the structure of the California flora. The randomization tests are based on comparing each cell’s observed statistic to a null distribution of values for that cell (in which taxon occurrences were distributed randomly across space, as described in Methods). Since the statistical cut-off is applied to each cell separately, the null expectation would be that 5% of pixels would fall outside the 95% confidence interval by chance alone, split evenly between high and low values. However, for PD, RPD, and RPE (as implemented in CANAPE), our results deviate substantially from the null expectation, with point and model-based results indicating that a far greater than the expected 5% proportion of grid cells statewide are significant. For example, CANAPE of the point data (Fig. 3) showed that about 25% of the grid cells are significant for RPE and are not evenly divided high and low, with about twice as many grid cells categorized as concentrations of paleo-endemism as compared to neo-endemism. These randomization results are only patterns, of course, and subject to different possible process interpretations. For example, because each cell’s taxon richness is held constant, randomization results for PD are a measure of phylogenetic dispersion; thus, the red cells in Fig. 2a and Fig. 6 show a pattern of phylogenetic clustering [34]. At least two non-mutually exclusive processes could generate the observed under-dispersed pattern: environmental filtering based on niche conservatism within clades, or effects of historical biogeography such as migration or extinction [35]. Data issues could also alter the expected frequency of PD at a regional scale, e.g., edge effects imposed by state borders, or herbarium collection campaigns conducted by botanists focused on a particular taxonomic group.

To aid with interpretation of these patterns, we describe them below in the context of each major Jepson ecoregion. These discussions are based on the results using the phylogram and herbarium point data; differences seen using the chronograms or modeled ranges are discussed in subsequent sections.

#### Northwestern California

The Northwestern California cells were generally high in observed richness and PD, medium for PE, and not statistically significant for PD, with some cells containing branches that are significantly longer than by chance in RPD. For all vascular plants, and angiosperms alone, much of the Klamath Ranges showed CANAPE cells of mixed endemism, while the North Coast Ranges north of Arcata Bay showed a concentration of paleo-endemism, and the coast and outermost Coast Ranges from about Salt Point (northern Sonoma County) to Bruhel Point (northern Mendocino County) showed mostly mixed endemism. Earlier suggestions of both concentrated paleo-endemism and neo-endemism along the North Coast [17, 31, 32] conform to our finding of either paleo-endemism or mixed endemism there. Concentrations of neo-endemism in the Klamath Ranges were seen in the gymnosperm analysis, while concentrations of mixed endemism were seen in the North Coast north of Cape Mendocino for both gymnosperms and pteridophytes. This region of the state has had a more equable climate through time [17], which has enabled it be a harbor for refugial taxa, some range-restricted, as highlighted here by the numerous significant CANAPE cells.

#### Cascade Ranges

The Cascade Ranges had high observed richness, PD, and endemism, but they were not significant in randomized PD. There were many cells with significant concentrations of long branches in the southern part of the region. Cells in the southern part of the Cascade Ranges appeared as paleo- or mixed endemism in the CANAPE analysis.

#### Great Valley

The Great Valley had low observed richness and endemism, as also found by Baldwin et al. [11] in their species-based, non-phylogenetic analysis of spatial patterns of diversity. Some San Joaquin Valley cells contained a significant under-representation of the phylogeny and significantly short branches. Many cells in the Sacramento Valley contained a significant over-representation of the phylogeny and significantly long branches. Both regions contained cells exhibiting either paleo-endemism or mixed endemism. In the turnover analysis, most Great Valley cells were grouped together and were most similar to the cells from the southern Channel Islands. The recovery of so much significant endemism in the Great Valley — especially paleo-endemism — is somewhat surprising, but in part may be due to our use of historical collections made before most of the area was cleared for agriculture. Alkaline/vernal pools of the Great Valley are notably rich in endemics and contribute substantially to the endemic flora of the region [36], where any remaining native vegetation should be of high conservation priority.

#### Sonoran Desert

The inferred concentrations of paleo-endemism in the Sonoran Desert using the unconstrained tree were partly due to the long-branched parasitic *Pilostyles* (Apodanthaceae). In the dated tree analysis, significantly high paleo-endemism was not inferred for the area because the branch length of *Pilostlyes* is highly reduced. Most of the Sonoran Desert demonstrated significant mixed endemism, but this desert extends into Mexico and Arizona. If the flora from regions outside California was included in the analysis, then significant endemism would be reduced but not eliminated, as can be seen when only taxa completely restricted to the state were analyzed (Additional file 4: Figure S3). From a conservation perspective, the potential drawbacks of using a political border to delimit a floristic study are offset because conservation decisions and funding are applied within political management units; thus, it is important to know local diversity and endemism patterns. A finer grained taxonomic sampling of spatial phylogenetic patterns in California’s Sonoran Desert is warranted to better understand endemism in the region in light of the much lower extent of cells of significantly high endemism in the species-based analysis of Baldwin et al. [11] than was found in the present study (Additional file 11: Figure S10).

#### Southwestern California

Only the southern Channel Islands and the immediate South Coast appeared as areas of significant endemism in Southwestern California in the CANAPE analysis. The islands have always been isolated from the mainland, which may have promoted the formation of new evolutionary lineages or the survival of older lineages (see [15, 17, 37]). Because our analysis is done at a more inclusive level than species, and the fact that the Channel Islands have a limited number of endemic taxa above the species level [38], we are not seeing the full amount of phylogenetic endemism, and more specifically neo-endemism, that would probably be detected in analyses at a shallower phylogenetic level. Nonetheless, detection of neo-endemic cells in the islands is consistent with recent evidence for evolutionary divergence there [15], in contrast to the older view that the island endemics are predominantly or entirely relictual [39]. The Transverse Ranges had high observed richness and endemism, as also found by Baldwin et al. [11] for their species-based analysis of spatial diversity patterns, yet were not significantly high in any of the randomization results.

#### Central Western California

The South Coast Ranges, including the Santa Lucia Range, did not contain significant centers of endemism in the vascular plant analysis (Fig. 3), but they stood out within the CA-FP for an under-representation of the phylogeny and significant concentrations of short branches, to an extent similar to that seen in the deserts (Fig. 2). Areas of significant endemism did appear in the gymnosperm analysis, resolved as neo- or mixed endemism in the phylogram and as paleo- or mixed endemism in the fully calibrated tree (Additional file 7: Figure S6). The Bay Area had high richness but only medium levels of endemism. It was not significant for randomized PD but did show some concentrations of significantly long branches in randomized RPD and showed some cells with mixed endemism in CANAPE. The Bay Area has previously been identified as a significant center of endemism including Mount Tamalpais, Mount Diablo, the Mount Hamilton Range, and the Santa Cruz Mountains [5], which were all areas of high WE based on the species-based non-phylogenetic analysis of Baldwin et al. [11] and were in part (e.g., Mount Tamalpais and the Santa Cruz Mountains) resolved as areas of high WE here (Additional file 11: Figure S10).

#### Sierra Nevada

The High Sierra Nevada showed high richness and endemism yet no significance for randomized PD. Some cells containing significantly long branches were observed in randomized RPD. A few cells showed mixed endemism and paleo-endemism in CANAPE. The areas of mixed endemism were in the vicinity of the Central and Southern Sierra Crest, including areas where Baldwin et al. [11] found significantly high species-level endemism in their non-phylogenetic assessment of spatial patterns of richness and endemism in the Californian vascular flora.

#### East of the Sierra Nevada

In the White-Inyo Range and Great Basin Desert areas there were medium levels of richness and endemism, while an under-representation of the phylogeny was detected in randomized PD, and significantly short branches were detected in randomized RPD. Areas of mixed endemism were more widespread than those of neo-endemism, which were concentrated in the White-Inyo Range and Sweetwater Mountains; these were also areas of significant endemism in the species-level study of Baldwin et al. [11]. Being near the Nevada border, one might suspect these centers of endemism are due to local occurrences of taxa that extend east into adjacent regions, as discussed above for the Sonoran Desert; however, they are still seen when analyzing only taxa restricted to California (Additional file 4: Figure S3).

#### Mojave Desert

Observed values differed greatly across the Mojave Desert, with, for example, richness and endemism being low in Death Valley, moderate in the Panamint Range, and elevated in the eastern desert mountains. All regions in the Mojave Desert had an under-representation of the phylogeny and significantly short branches, as detected in randomized PD and RPD, respectively. The branches were short in Death Valley and the eastern desert mountains but long in the Panamint Range. Many eastern Mojave cells showed mixed endemism or neo-endemism in CANAPE. Kraft et al. [32] also found a high concentration of young endemics in the Mojave Desert based on their analysis of a select group of neo-endemic lineages.

#### Modoc Plateau

Low observed richness and endemism were seen in the Modoc Plateau except for the Warner Mountains, as also seen in the species-level non-phylogenetic analysis of Baldwin et al. [11]. The Warner Mountains have been long recognized for having a diverse flora composed of taxa representative of different source areas [40]. Many cells in the Modoc Plateau had an under-representation of the phylogeny, and some had significantly short branches, as detected in randomized PD and RPD, respectively. This area showed concentrations of all types of endemism, but not when only taxa restricted to California were analyzed. As for California’s Sonoran Desert, much of the diversity of lineages in the Modoc Plateau that are range-restricted within the state occurs more widely outside California.

#### Climate and endemism

One putative explanation for areas of endemism is that they have been climatically stable or had low climate velocities over a long period of time, with mountains or ocean currents forming refugial conditions, as has recently been supported at a global scale [41, 42]. However, those conclusions concern areas of high absolute endemism and do not necessarily pertain to areas that have higher relative endemism than expected by chance. Our CANAPE analyses using herbarium point data or the distance hybrid range models did not highlight the High Sierra Nevada or the central Pacific coast as areas of significantly high phylogenetic endemism, but the unconstrained Maxent range models did.

The comparison of four climatic variables with the significant CANAPE grid cells from the analysis using non-modeled herbarium points (Fig. 3) suggests that most of these cells occur in areas that are low in precipitation, i.e., desert or semi-arid environments. Stebbins [43] argued for aridity as a stimulus for plant evolution, but spatial analyses indicating a positive association between aridity and floristic endemism have been lacking until now. Clustering of cells with similar climate values (Fig. 3e) suggests that the phylogenetic composition of groups could be influenced more generally by climate, especially precipitation.

Other climatic variables showed less apparent correlation with significant endemism than did precipitation (Additional file 10: Figure S9), which leads to the question of whether precipitation or lack thereof contributes toward conserving or creating endemism — or both — and, if so, how. Stebbins [43] maintained that aridity should lead to more rapid evolution because topography, soils, and other environmental variables would affect the vegetation and habitat characteristics to a greater degree than in more mesic environments; that is, community or habitat diversity should be greater in drier environments even if plant diversity in any particular habitat is low, and those conditions should lead to more speciation (and extinction). Variables such as topography and edaphic conditions were not analyzed in this study. Correlations between these two variables and endemism may be likely, but the 15 × 15 km resolution of our study is unfavorable for detecting such a correlation because many cells would most likely be highly heterogeneous, especially for soil, at this grid scale. One way to overcome poor resolution is to model plant ranges so that a finer distributional resolution can be achieved. However, as discussed below, using modeled ranges can have a dramatic effect on CANAPE results, and may be subject to a confounding effect if the same variables used to produce the ranges are then being studied for relationships between climate and range size (i.e., endemism). A more productive option for future finer scale comparisons between patterns of relative endemism and environmental variables would be more field studies.

### Methodological findings

#### The effects of range modeling

The overall effect of both niche-modeling approaches applied here was a drastic increase in observed taxon richness (Additional file 9: Figure S8B, C) and decrease in observed endemism, with smoothing of both across the landscape, as estimated taxon ranges became notably larger and more continuous (Fig. 7) than those based on herbarium point records. In addition, geographic concentrations of significant cells detected in PD, RPD (Fig. 6), and CANAPE (Fig. 5) were in some cases larger, yet in other cases smaller or absent, as compared to the results using the occurrence data. The discrepancies may reflect a combination of some ranges being under-represented by herbarium specimens and others over-represented by climatic niche models. If modeling artificially increases the apparent range size of taxa, endemism decreases in general and localized concentrations of endemism may become more difficult to find. Range limits of many species are governed by factors other than macroclimate, e.g., soil specialization, occupation of specialized microenvironments, biotic interactions with predators or pollinators, geographic barriers to dispersal, or historical accidents wiping out populations [44, 45], and modeling should be extended to include as many of these factors as possible [46].

We hypothesize that the most range-restricted (i.e., highly endemic) plants are particularly unlikely to be limited in their distribution primarily by macroenvironmental factors, and thus may be especially overestimated by species distribution modeling, thereby causing significant patterns of endemism to be missed. There is some support for this hypothesis in our data (see Fig. 7): modeled range size does increase proportionately more in taxa that have geographically narrow collection-based ranges, particularly for the standard Maxent models without distance constraints. Further investigation of this hypothesis is an important goal for future studies.

Of the two modeling methods applied here, endemism patterns were less affected by the distance hybrid method than by the standard Maxent method, although the patterns still appeared different from the CANAPE results found using herbarium spatial data (Fig. 5). These differences are sufficient to warrant caution in applying CANAPE, which is sensitive to the estimates of taxon ranges. Modeling is one of the only available approaches to predict what may happen to diversity and endemism in the future [2, 12], and it needs to be further investigated and improved [47]. Likewise, gaps need to be filled in our knowledge of plant distributions as documented by herbarium specimens; further targeted fieldwork is essential.

#### Chronograms versus phylograms

Both approaches to assessing branch lengths on trees make assumptions. They both assume that the genes that were studied are representative of other genes in the genome. And both may be affected by rate heterogeneity, or by changes in extinction and speciation rates, in different parts of the tree. For example, it is widely observed that growth form affects molecular evolutionary rates, with annuals tending to evolve faster than woody plants [48]. Thus, interpretations of neo- or paleo-endemism have to be tempered by consideration of these possible biases.

Deciding whether to base conservation decisions on chronograms or phylograms requires some reflection on the goals of conservation. Areas of concentrated neo- and paleo-endemism found with CANAPE using a phylogram reflect the occurrence of range-restricted long or short branches defined by genetic differences, which are likely to correlate with other features of the organisms: *feature diversity* [49]. If the goal of conservation is to conserve genotypic and phenotypic diversity, this approach may be the method of choice. On the other hand, the importance of areas that contain organisms with highly modified genotypes and phenotypes (e.g., parasites) might be overstated using such data, if conserving genetic/feature diversity is not the goal. Neo- and paleo-endemism areas identified with CANAPE using a chronogram reflect range-restricted long or short branches defined by *time*. If the goal of conservation is to conserve evolutionary history, this approach may be the method of choice.

Fortunately, it is evident from comparing our CANAPE results using the phylogram with those using two different chronograms that the locations of centers of significant PE are relatively stable regardless of whether a phylogram, minimally calibrated tree, or fully calibrated tree was used (Fig. 4). What did change was the interpretation of endemism for some of the significant CANAPE cells. For example, the area interpreted in the vascular plant analysis as containing concentrated paleo-endemism in the Sonoran Desert changed to an interpretation of mixed endemism once a chronogram was used. In the gymnosperm analysis, the cluster of cells interpreted as containing concentrated neo-endemism in the Monterey and Big Sur region changed to an interpretation of paleo-endemism with the fully calibrated phylogeny.

Thus, the identification of significant endemism cells is not as dependent on tree topology or branch lengths in the tree as it is on the spatial occurrence data. Further evidence of the robustness of our analyses to phylogenetic uncertainty is the CANAPE findings using 10 different maximum likelihood topologies (Additional file 8: Figure S7). Subtle differences can be found among the results from the 10 topologies, but they may be attributable to the randomization process as much as to tree uncertainty. Major topological changes could of course affect patterns of significance, but the difference in tree topology would need to be large to cause much change. Tree uncertainty usually involves very short branches that switch to a different branching pattern in a set of nearly optimal trees; the amount of PD or PE contributed by such branches is minimal [2].

## Conclusions

This study demonstrates the importance of examining diversity and endemism from a phylogenetic perspective, to better understand the evolutionary composition and conservation value of different areas within a biotic region. In particular, use of derived metrics that examine the significance of branch length differences across the region adds a new dimension to California floristics that, in comparison with climatic data, helps to illuminate endemism patterns and their causes. The concentration of short branches and neo-endemism in more arid regions of California, including the Great Basin, Mojave Desert, and South Coast Ranges, reinforces and extends preliminary findings suggesting that the youngest Californian neo-endemics are mostly outside the CA-FP [32] and ideas of Stebbins [43] about aridity as an evolutionary stimulus. Robustness of spatial phylogenetics to topological uncertainty and temporal calibration of trees increases confidence in the results. However, sensitivity to use of occurrence data versus modeled ranges as shown here indicates that special attention toward improving geographic distributional data should be top priority for advancing understanding of spatial patterns of biodiversity.

## Methods

### Spatial records

California was selected as our study region because of spatial data availability, the ability to examine most of the CA-FP in a broader spatial context, and California’s status as a political unit in which conservation decisions and funding are applied. As outlined in Baldwin et al. [11], spatial coordinate information for native California vascular plants was downloaded from the following five sources for herbarium records: Consortium of California Herbaria (http://ucjeps.berkeley.edu/consortium), Consortium of Pacific Northwest Herbaria (http://www.pnwherbaria.org), Australia’s Virtual Herbarium (http://avh.ala.org.au/), Canadensys (http://community.canadensys.net/), and GBIF (http://www.gbif.org), and subsequently cleaned for taxonomic and spatial consistency. We began with the species-level spatial dataset and combined the species into OTUs (Additional file 12: Table S2). For some OTUs, additional records could be added that were identified only to genus in the dataset used by Baldwin et al. [11]. The final spatial dataset had 1,395,079 records and is deposited in the University of Caifornia Berkeley (UC Berkeley) DASH repository [50].

### OTU definition

Instead of using a uniform taxonomic rank (such as species or genus) to define OTUs, as is typically done, we took a novel, pragmatic phylogenetic approach to define OTUs for this study. The goal was to define monophyletic OTUs at the finest scale possible given data availability and current understanding of the evolutionary relationships of Californian plant lineages. Using all 5258 species and 993 genera of native California vascular plants described in *The Jepson Manual* [13] or subsequently recognized by the Jepson Flora Project [51] as a starting point, a thorough literature search was undertaken to find molecular phylogenetic studies that had included California taxa. Genera were split into finer level OTUs if robust evidence existed for monophyly of subclades and representative DNA data either were available in GenBank or could be generated within the scope of the present project (see next section). Genera were lumped in a few cases if recent evidence showed that one is nested in another. In total, 1083 OTUs were defined to include the 5258 binomials (Additional file 12: Table S2 details the OTU to which each binomial was assigned). The 1083 OTUs ranged in size from 1 to 155 binomial taxa (median = 2); 948 OTUs contained 10 or fewer binomial taxa.

### DNA sequence data assembly

Sequences were downloaded from GenBank [52] using Matrix Maker ([53] https://github.com/wf8/matrixmaker), a Python utility that uses both currently accepted and synonymous names to mine GenBank and assemble sequence matrices. Nine genetic markers were targeted: nuclear ribosomal (nrDNA) 18S and ITS [ITS-1, 5.8S, and ITS-2]; plastid (cpDNA) *ndh*F, *atp*B, *mat*K, *rbc*L, and *trn*L-*trn*F; and mitochondrial ribosomal *rps*4 and *mat*R (see Additional file 13: Table S3 for accessions). In total, 3366 sequences were downloaded from GenBank with representatives from 90% of California native genera and 45% of native California binomials. Every OTU except for three was represented using sequences exclusively from native Californian species. *Bistorta*, *Chrysosplenium*, and *Crocanthemum* were represented by sequences from non-Californian taxa.

New sequences were generated for the OTUs that were not represented by a sequence in GenBank. Three DNA regions (nrDNA: ITS; cpDNA: *rbc*L *mat*K) were sequenced for this study; sequences were recovered for 342 OTUs, and 879 new sequences were deposited in GenBank. Voucher information and GenBank accession numbers are compiled in Additional file 13: Table S3. Total genomic DNA was isolated from herbarium specimens or fresh leaf tissue using a DNeasy plant extraction kit (Qiagen, Hilden, Germany) or PowerPlant Pro DNA Isolation Kit (MoBio Laboratories, Carlsbad, CA, USA) according to the manufacturers’ protocols. Primer information and details of PCR amplification for all markers are summarized in Table 1. The amplicons were purified with ExoSAP-IT (Affymetrix, Santa Clara, CA, USA) following the manufacturer’s protocol with minor modifications. Cycle sequencing reactions were carried out at the UC Berkeley DNA Sequencing Facility, using the same primers as were used for the PCR amplifications. Forward and reverse chromatogram sequences were manually reviewed, edited, and assembled using Geneious version 6.1.8 (http://www.geneious.com, [54]). In order to detect and remove any potential sequence contamination, the identity of each generated sequence was checked using BLAST (Basic Local Alignment Search Tool, National Center for Biotechnology Information, https://blast.ncbi.nlm.nih.gov). References [55–59] were the sources for the information in Table 1.

**Table 1:** Oligonucleotide primers used to amplify and sequence the *rbc*L, *ma*tK, and ITS markers and their associated PCR thermocycling regimes

Marker	Primer	Source	PCR conditions
*rbc*L	aF	Pryer et al., 2001 [55]	5 min of initial denaturation at 80 °C; 30 cycles: 95 °C 1 min, 60 °C 1 min, 65 °C 2 min; final extension 5 min
	1379R	Pryer et al., 2001 [55]	
*mat*K	matK-xf	Ford et al., 2009 [56]	5 min of initial denaturation at 95 °C; 35 cycles: 95 °C 30 s, 49 °C 1 min, 72 °C 1 min 40 s; final extension 7 min
	matK-MALP	Dunning and Savolainen, 2010 [57]	
ITS	ITS5a	Stanford et al., 2000 [58]	6 min of initial denaturation at 95 °C; 35 cycles: 95 °C 30 s, 49 °C 1 min, 72 °C 1 min 40 s; final extension 7 min
	ITS4	White et al., 1990 [59]	

### Phylogenetic analyses

DNA sequence alignments were made using MAFFT [60] with default settings except for the “--adjustdirection” option, which checked for correct sequence polarity. The concatenated 26,993 base pair sequence matrix and our trees are available as a Nexus file at the online UC Berkeley DASH repository [61]. We performed maximum likelihood phylogenetic analyses using RAxML 8.2.9 [62] on the CIPRES computing infrastructure [63]. We applied the general time-reversible (GTR) + CAT model [64] of nucleotide substitution with 25 rate categories independently to each of the nine gene partitions, performing tree searches with a randomized stepwise addition order maximum parsimony starting tree and 1000 rapid bootstrap replicates [65]. The analyses utilized a constraint tree that enforced the following high-level relationships [66]: Isoëtales and Selaginellales were made a monophyletic clade sister to the Lycopodiales, the Lycopodiophyta were set to be monophyletic and sister to the Pteridophyta plus spermatophytes, and the Pteridophyta and spermatophytes were each constrained to be monophyletic and sister to one another. We replicated the RAxML analysis using 10 different random number seed values and compared the resulting 10 maximum likelihood trees by their log-likelihood values and Robinson-Foulds distances [67].

### Molecular dating

We produced time-calibrated chronograms using penalized likelihood [68] as implemented in r8s [69] using the truncated Newton method and a smoothing parameter of 1000. Fossil calibrations were taken from Magallón et al. [70, 71], and two different calibration schemes were utilized: the first used only a root age for all vascular plants, and the second used 55 calibrations scattered across the whole phylogeny (a list of node calibrations is provided in Additional file 14: Table S4). Using the latter calibration, the stem age of our OTUs (length of the terminal branch) ranged from 0.2 to 382 my (million years) (median = 27 my).

### Modeled ranges using macroclimatic variables

The species distribution modeling algorithm Maxent [72] was used to model the range of each Californian species, using the cleaned species-level spatial dataset from Baldwin et al. [11], deposited in the UC Berkeley DASH repository [73]. Models were fit at the species level since gene flow barriers among species mean that climatic niches are relatively cohesive within species but not within OTUs; each OTU’s range was then taken to be the union of the ranges of its included species. Models were fit using four predictor variables representing major energy- and water-related variables known to be important for California plant distributions: climatic water deficit, annual precipitation, mean summer maximum temperature (June–August), and mean winter minimum temperature (December–February). All climate data were obtained from the California 2014 Basin Characterization Model (1951–1980 averages [74]) at 270-m resolution and up-scaled to 810-m resolution to reflect spatial uncertainty in specimen collection localities.

The modeling domain was restricted to the state of California to match the spatial coverage of the herbarium dataset. This same domain was used for all species. While this political boundary unavoidably excluded the out-of-state distributions of non-endemic species, the effect on predicted distributions within California is probably minimal, since neighboring regions have climates relatively dissimilar from most of California (making them uninformative for prediction within California) and since California includes a large diversity of internal climate gradients (offering abundant data for inference of climate-range relationships).

To reduce spatial sampling bias, we down-sampled records to one per species per 810-m grid cell, and then used climate values from 10,000 randomly sampled species records across California as background data to account for sampling intensity. Default Maxent parameters were used with linear and quadratic terms and no threshold or hinge features to reduce overfitting of the models. Predicted suitability values were converted to binary presence-absence maps using the “sum of specificity and sensitivity” threshold, which gives equal weight to false presences and absences. The range of each OTU was defined as the union of the thresholded ranges of its constituent species, with a mean of 4.8 species per OTU. All modeled ranges and the scripts used to make them are available at the online DASH repository [75]. The average species model was based on 176 occurrence records after down-sampling, and fit the training data well with an area under the curve (AUC) of 0.913. The number of records, AUC, and OTU membership for each species are listed in Additional file 15: Table S5.

In addition to the standard Maxent modeling approach described above, which estimates an unconstrained distribution of climatically suitable habitat, we also used a second “distance hybrid” approach to model the area for each species that was both climatically suitable and geographically close to observed occurrences. The distance constraint arguably increases realism by accounting for spatial processes such as dispersal limitation and metapopulation dynamics. For each species, grid cells were assigned weights from 0 to 1 based on distance to the nearest occurrence record, using a Gaussian distance decay function with a sigma of 50 km. To generate the hybrid model, this weight was multiplied by the continuous Maxent suitability prediction before thresholding. The effect of this distance constraint was that 50% of predicted presences fell within 15 km of occurrences and 99% fell within 70 km. In the unconstrained models, we found that 50% of predicted presences fell within 25 km and 99% within 350 km of observed locations.

### Spatial phylogenetic analyses

The spatial data (composed of georeferenced herbarium specimens) were first compared against the phylogram (i.e., the topology with uncalibrated branch lengths inferred in the ML analysis) using Biodiverse [76]. The spatial coordinate information for each record was converted to presence within 15 × 15 km grid cells (Albers equal-area, EPSG:3310), a size justified in Baldwin et al. [11]. An OTU was recorded as present in a grid cell if one or more species were present. Using the methods and metrics described by Mishler et al. [1] and Thornhill et al. [3], observed patterns of taxon (OTU) richness (TR), weighted endemism of taxa (WE), phylogenetic diversity (PD), and phylogenetic endemism (PE) were mapped. Spatial randomizations were run on the dataset and compared against the observed results to estimate significance of PD, RPD, and RPE as applied in CANAPE. These null distributions were generated by repeatedly reshuffling the identities of the taxa found in each grid cell from a statewide taxon occurrence pool, holding constant the total taxa per cell and the total cells per taxon. This null model assumes that a taxon’s occurrences display no spatial autocorrelation. The randomizations and figures were generated using an R Biodiverse pipeline (https://github.com/NunzioKnerr/biodiverse_pipeline). The dataset was re-analyzed with an appropriately trimmed phylogeny for four subsets of the OTUs: angiosperms, gymnosperms, pteridophytes, and taxa completely restricted to California.

To determine the effect of species distribution modeling on patterns of significance, further analyses were performed for all vascular plants using the Maxent and distance hybrid distribution models, which we aggregated from 810-m to 15-km resolution in the same fashion as specimen point data. To determine the effects on significance patterns of phylogenetic uncertainty, we analyzed the vascular plant herbarium record dataset using 10 independent RAxML phylograms with different log-likelihood values. Finally, to determine the effects on significance patterns of using dated phylogenies, we repeated analysis of the vascular plant herbarium record dataset against two different chronograms (i.e., topologies with time calibrations as described above).

### Turnover among cells showing significant PE

We investigated turnover among significant CANAPE cells (resulting from the analysis using the phylogram and vascular plant herbarium record dataset) by clustering them using the Unweighted Pair Group Method with Arithmetic Mean (UPGMA), using the range-weighted phylogenetic metric [77], as implemented in Biodiverse. This analysis highlights geographic regions within which the evolutionary makeup of the endemic flora is relatively homogeneous. The approach looks pairwise at the length of branches shared between grid cells, where each branch length has been divided by its range; shared range-restricted branches thus count more toward similarity than shared widespread branches.

### Climate-phylodiversity relationships

Climatic predictors of floristic phylodiversity were examined by comparing spatial patterns in four climate variables (those detailed above) with our results for PD, RPD, and RPE. We also assessed relationships between these variables and significance levels from the randomization test as well as cluster results from the turnover analysis. For simplicity, climate was compared only to results for the phylogram tree topology, and to avoid circularity, climate was not compared to results for species distribution models.

## Additional files

Additional file 1: Table S1. Robinson-Foulds distances between the ten California plant phylogenies produced with RAxML. (CSV 464 bytes)

Additional file 2: Figure S1. Phylogeny of 1083 clades representing all vascular plants in California. The tree is mapped with the orders from APG III to show that all orders are monophyletic. (TIF 1268 kb)

Additional file 3: Figure S2. Bootstrap support of the clade phylogeny. Phylogeny of 1083 clades representing all vascular plants in California, with branches colored according to their bootstrap value. (TIF 13160 kb)

Additional file 4: Figure S3. Subset analyses of California plants. Comparison between the observed PD, randomized PD, randomized RPD, and CANAPE results for all vascular flora and four subsets of Californian plants. Angiosperms alone showed a similar result to the vascular plant analysis, while the others differed. (TIF 19879 kb)

Additional file 5: Figure S4. The various phylogenetic topologies used in the derived metrics RPD and RPE. (a) Original tree (used in the numerator of RPD) and its range-weighted equivalent (used in the numerator of RPE) for the non-calibrated tree (*left*), root-calibrated tree (*center*), and fully calibrated tree (*right*). (b) The equal branch length comparison tree (used in the denominator of RPD) and its range-weighted equivalent (used in the denominator of RPE). (TIF 1709 kb)

Additional file 6: Figure S5. A comparison of randomized PD and randomized RPD, for all vascular plants using an uncalibrated, root-calibrated, and fully calibrated phylogeny. The location of significant cells remains relatively constant for all three tree types with the notable exceptions of new significant cells of PD and RPD appearing in the northwest in the calibrated trees. (TIF 5879 kb)

Additional file 7: Figure S6. A comparison of CANAPE analyses. CANAPE results for uncalibrated, root-calibrated, and fully calibrated phylogeny for all vascular flora and four subsets of Californian plants. (TIF 10767 kb)

Additional file 8: Figure S7. The effect of phylogenetic uncertainty. A comparison of the CANAPE results for all vascular plants using the topologies from 10 different RAxML runs. (TIF 416 kb)

Additional file 9: Figure S8. A comparison of richness of all vascular Californian. Analyses measured with (a) the point-occurrence data, (b) distance hybrid modeled ranges, and (c) binary Maxent clade modeled ranges. (TIF 1680 kb)

Additional file 10: Figure S9. Plots of four climate variables. Colored with significance for PD (Fig. 2a), RPD (Fig. 2c), and RPE (from CANAPE; Fig. 3a); and by their cluster in the turnover analysis (Fig. 3c and e). (TIF 15703 kb)

Additional file 11: Figure S10. Diversity analyses measured using terminal clades (OTUs) as taxa (i.e., without using a phylogeny). Clockwise from left, Richness, weighted endemism (WE), controlled weighted endemism (CWE), and significantly high endemism following randomization (Rand). (TIF 1622 kb)

Additional file 12: Table S2. A table showing where each native species of California was assigned to an OTU. (XLSX 194 kb)

Additional file 13: Table S3. The species and GenBank accessions used to represent each OTU. (XLSX 126 kb)

Additional file 14: Table S4. All calibrations used to date the Californian plant phylogeny. (XLSX 129 kb)

Additional file 15: Table S5. The number of records, area under the curve (AUC), and OTU membership for each native Californian plant species. (XLSX 230 kb)

## Abbreviations

CA-FP: California Floristic Province
CANAPE: Categorical Analysis of Neo- and Paleo-Endemism
OTU: Operational Taxonomic Unit
PD: Phylogenetic Diversity
PE: Phylogenetic Endemism
RPD: Relative Phylogenetic Diversity
RPE: Relative Phylogenetic Endemism
TR: Taxon Richness
WE: Weighted Endemism
