# Supplementary figures and images for: Spatial phylogenetics of the native California flora
Source: BMC Biol. 2017 Oct 26;15:96. doi: 10.1186/s12915-017-0435-x (PMC5658987; doi:10.1186/s12915-017-0435-x)

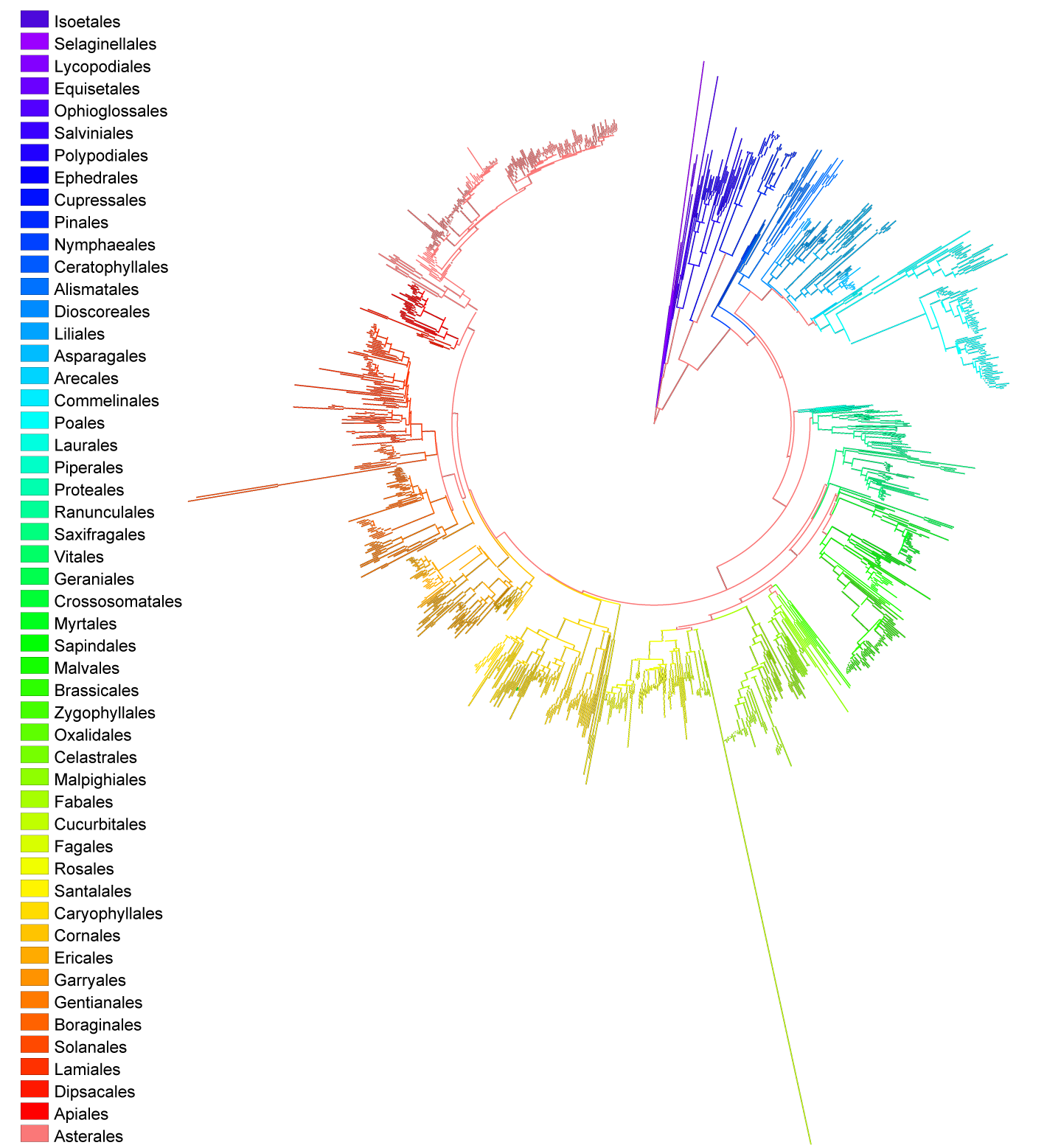

Supplement: Supplementary file 2 — Phylogeny of 1083 clades representing all vascular plants in California. The tree is mapped with the orders from APG III to show that all orders are monophyletic. (TIF 1268 kb) [file 12915_2017_435_MOESM2_ESM.tif]

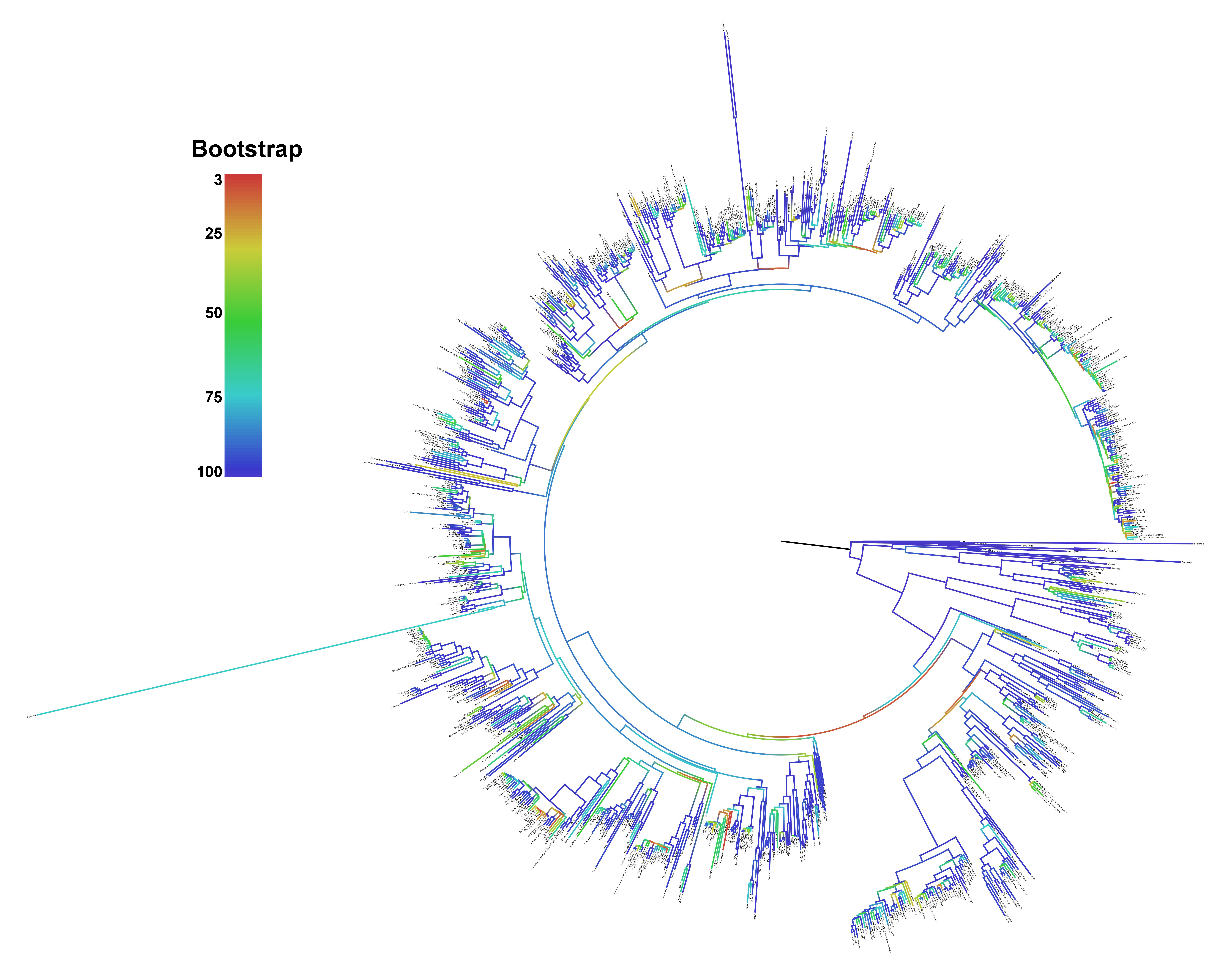

Supplement: Supplementary file 3 — Bootstrap support of the clade phylogeny. Phylogeny of 1083 clades representing all vascular plants in California, with branches colored according to their bootstrap value. (TIF 13160 kb) [file 12915_2017_435_MOESM3_ESM.tif]

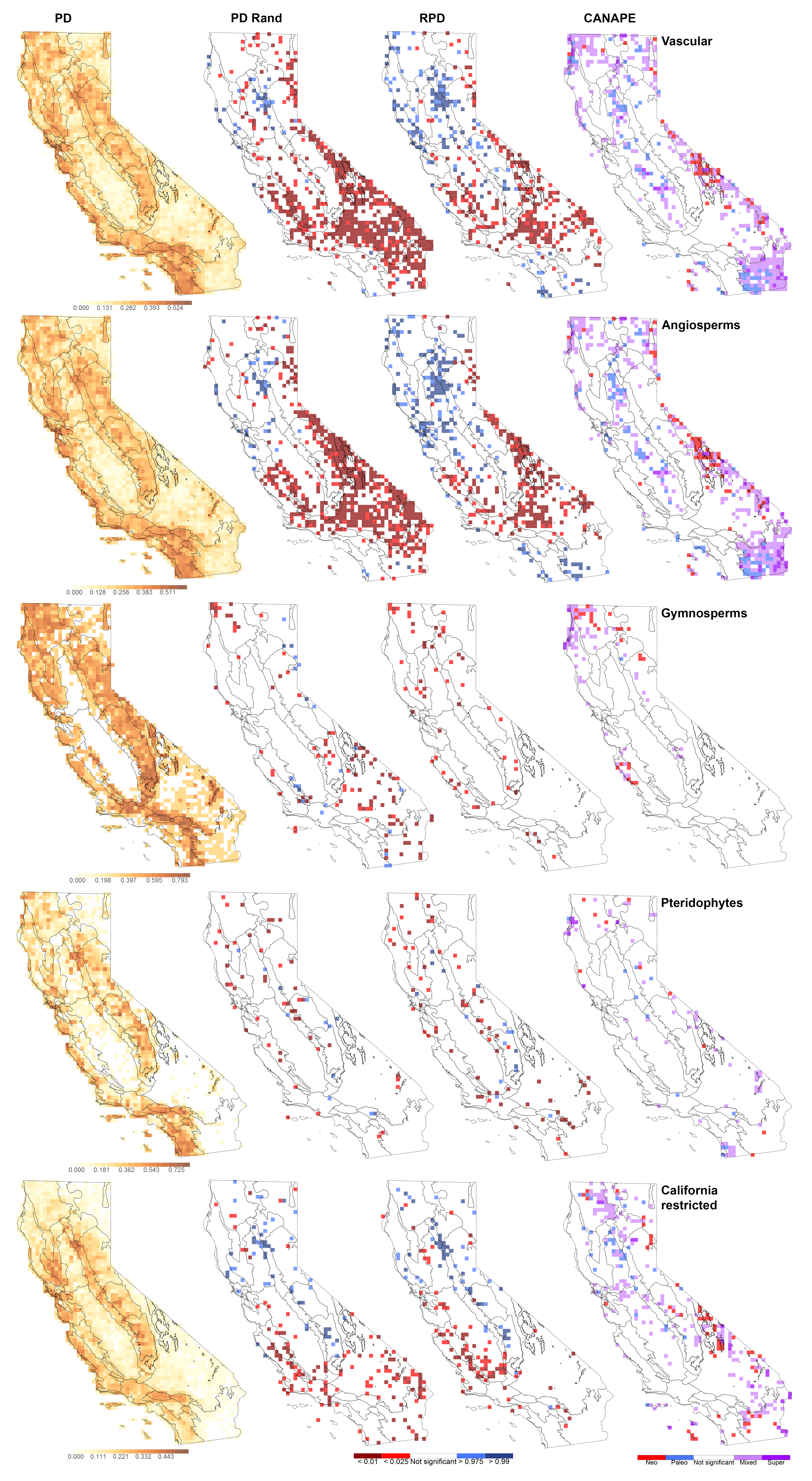

Supplement: Supplementary file 4 — Subset analyses of California plants. Comparison between the observed PD, randomized PD, randomized RPD, and CANAPE results for all vascular flora and four subsets of Californian plants. Angiosperms alone showed a similar result to the vascular plant analysis, while the others differed. (TIF 19879 kb) [file 12915_2017_435_MOESM4_ESM.tif]

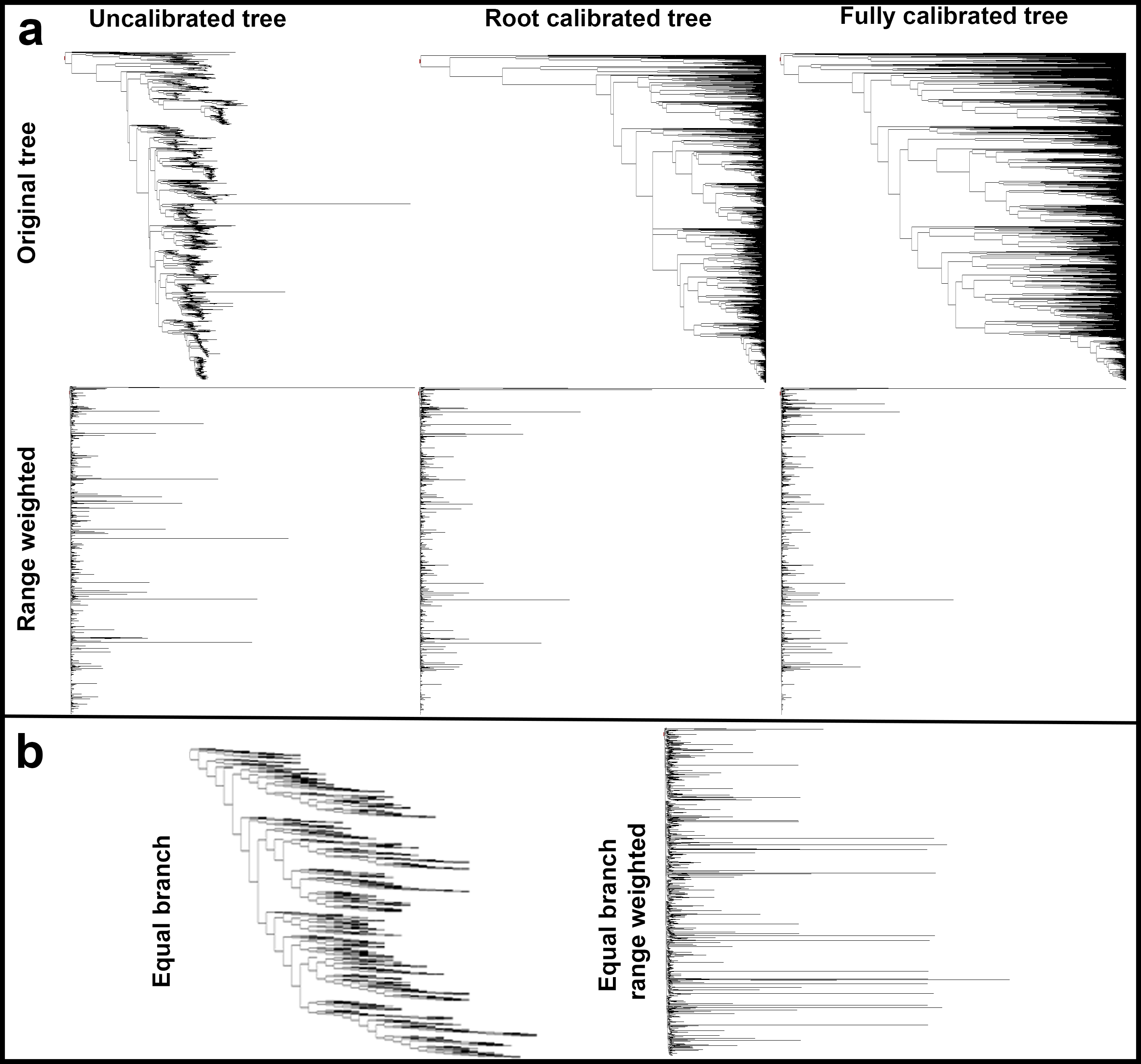

Supplement: Supplementary file 5 — The various phylogenetic topologies used in the derived metrics RPD and RPE. (a) Original tree (used in the numerator of RPD) and its range-weighted equivalent (used in the numerator of RPE) for the non-calibrated tree (left), root-calibrated tree (center), and fully calibrated tree (right). (b) The equal branch length comparison tree (used in the denominator of RPD) and its range-weighted equivalent (used in the denominator of RPE). (TIF 1709 kb) [file 12915_2017_435_MOESM5_ESM.tif]

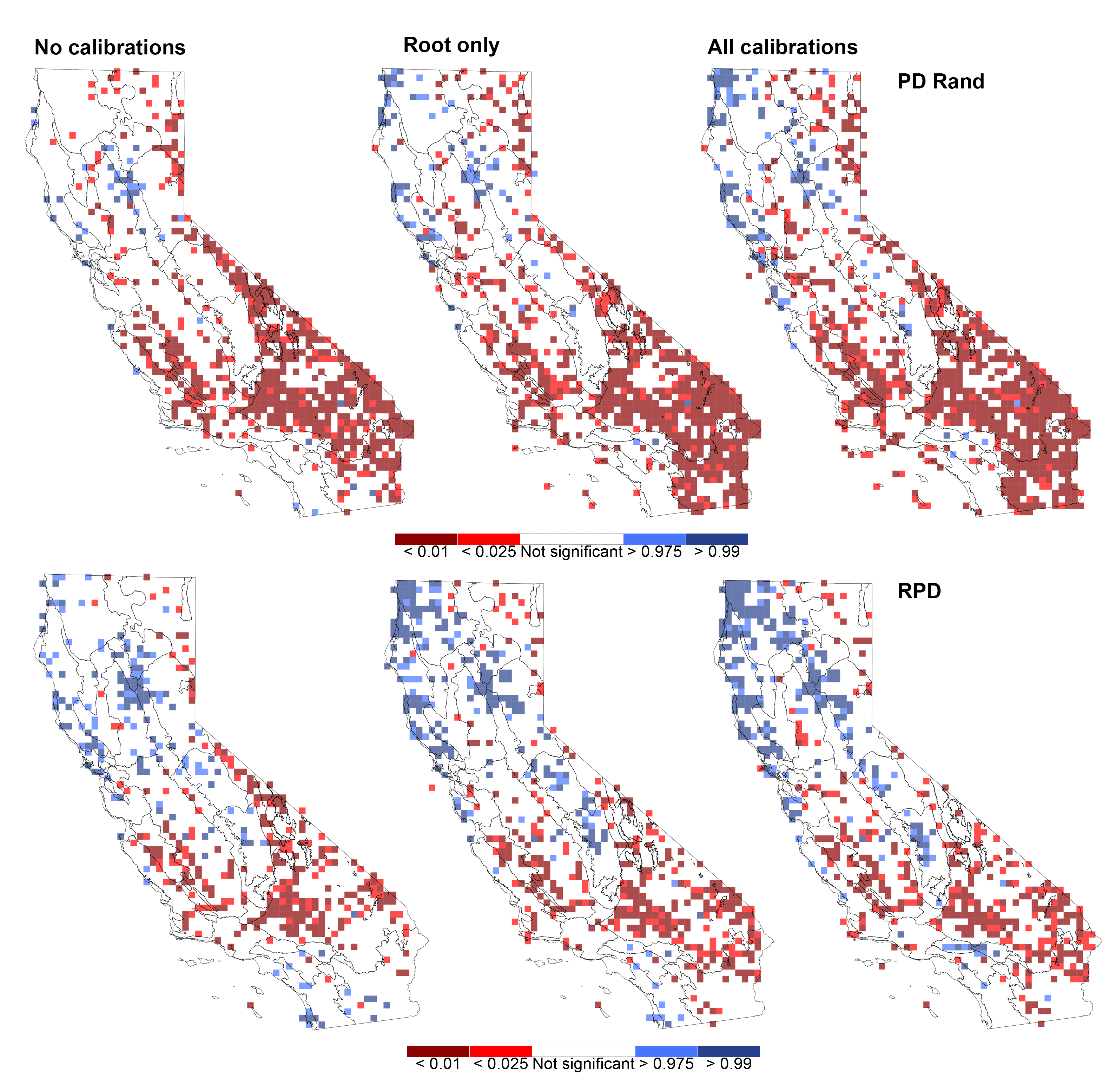

Supplement: Supplementary file 6 — A comparison of randomized PD and randomized RPD, for all vascular plants using an uncalibrated, root-calibrated, and fully calibrated phylogeny. The location of significant cells remains relatively constant for all three tree types with the notable exceptions of new significant cells of PD and RPD appearing in the northwest in the calibrated trees. (TIF 5879 kb) [file 12915_2017_435_MOESM6_ESM.tif]

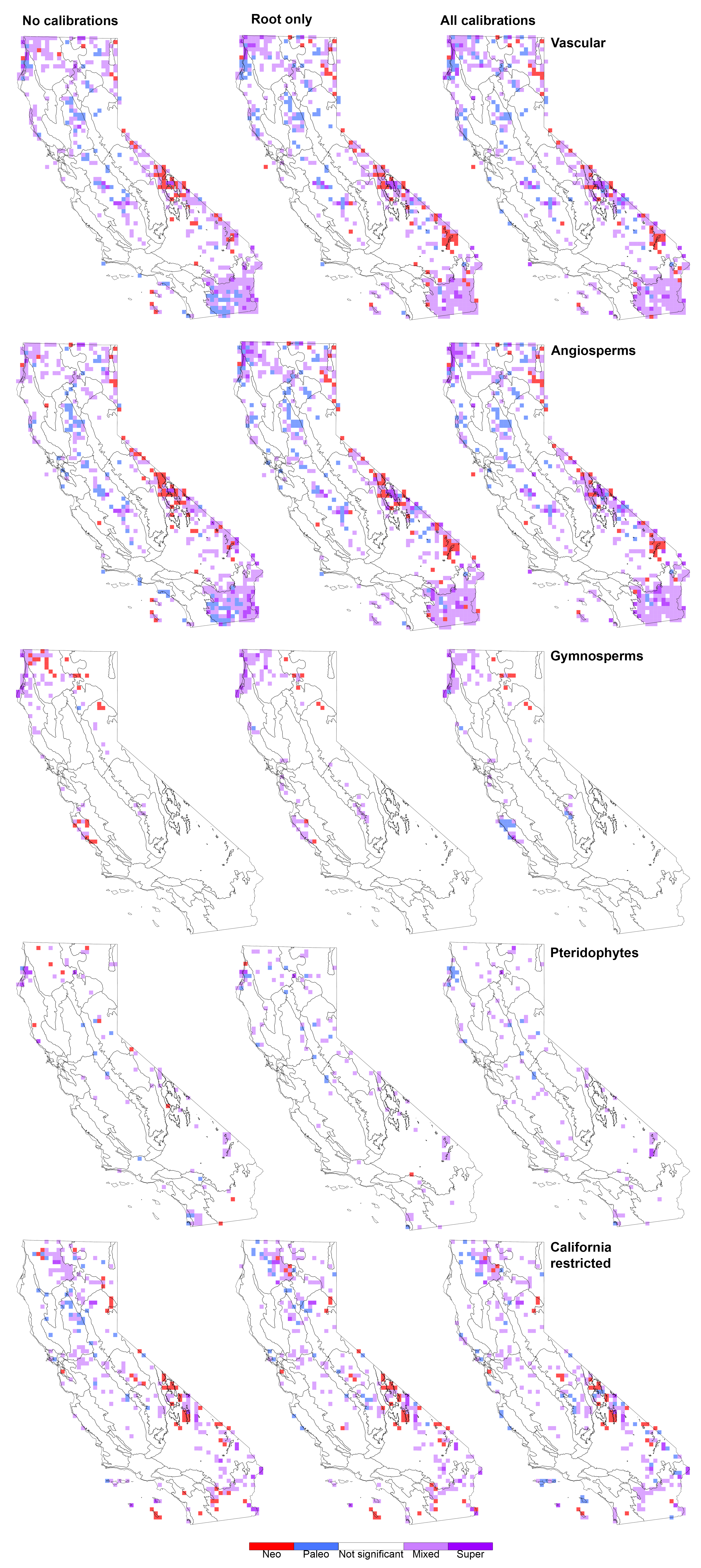

Supplement: Supplementary file 7 — A comparison of CANAPE analyses. CANAPE results for uncalibrated, root-calibrated, and fully calibrated phylogeny for all vascular flora and four subsets of Californian plants. (TIF 10767 kb) [file 12915_2017_435_MOESM7_ESM.tif]

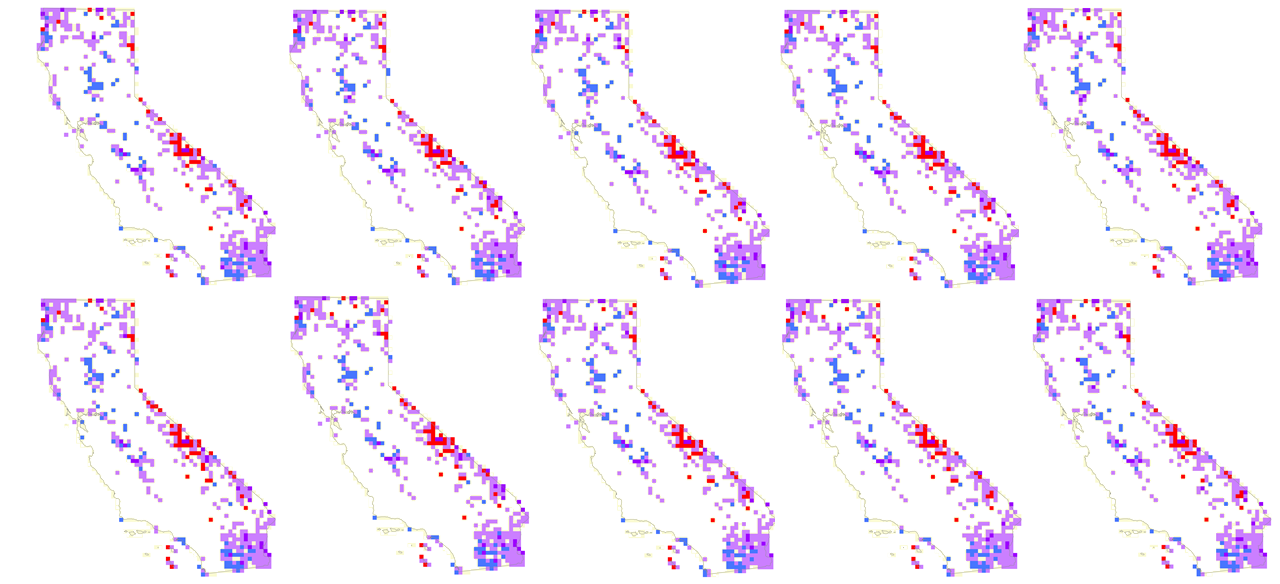

Supplement: Supplementary file 8 — The effect of phylogenetic uncertainty. A comparison of the CANAPE results for all vascular plants using the topologies from 10 different RAxML runs. (TIF 416 kb) [file 12915_2017_435_MOESM8_ESM.tif]

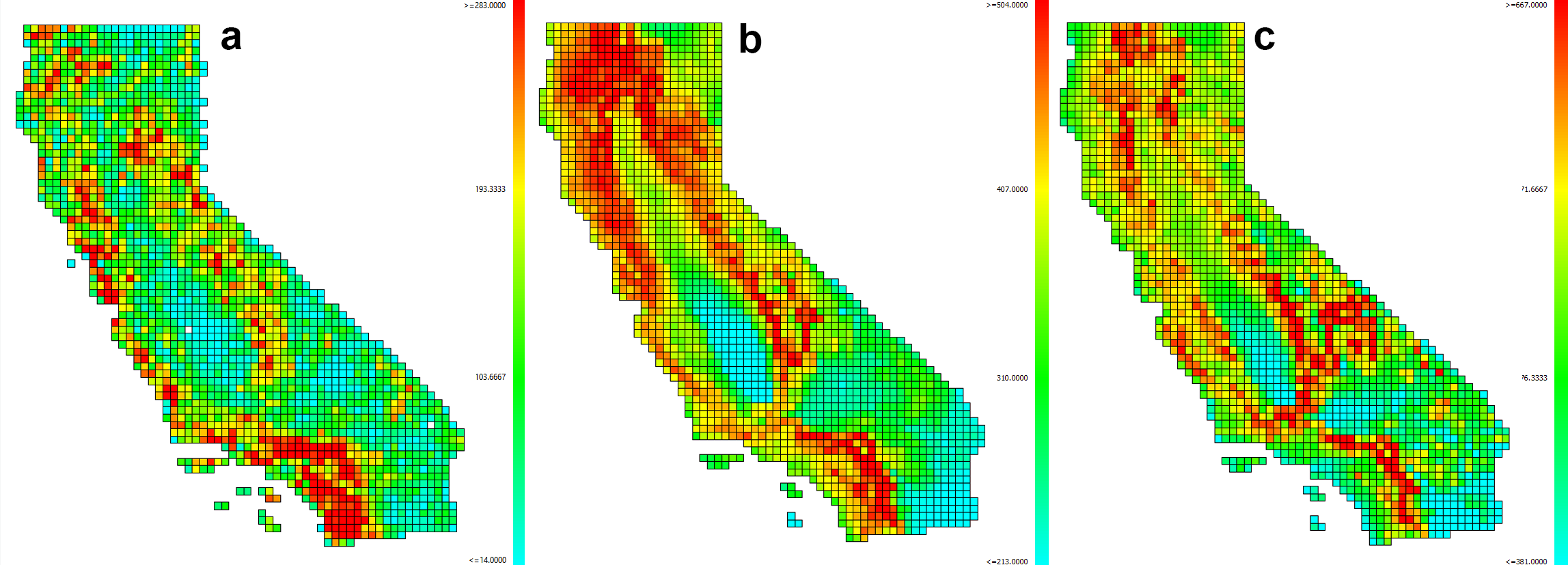

Supplement: Supplementary file 9 — A comparison of richness of all vascular Californian. Analyses measured with (a) the point-occurrence data, (b) distance hybrid modeled ranges, and (c) binary Maxent clade modeled ranges. (TIF 1680 kb) [file 12915_2017_435_MOESM9_ESM.tif]

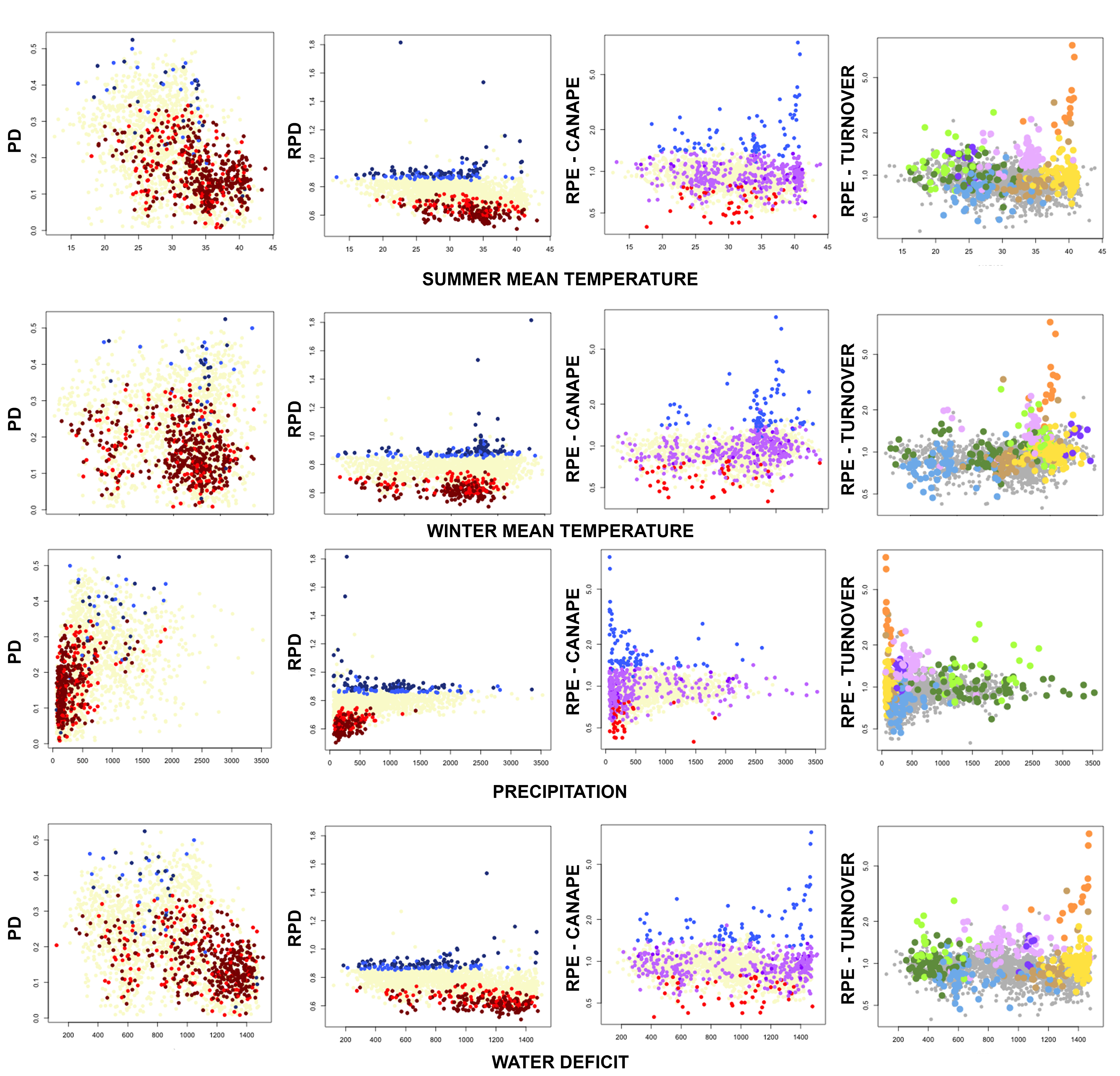

Supplement: Supplementary file 10 — Plots of four climate variables. Colored with significance for PD (Fig. 2a), RPD (Fig. 2c), and RPE (from CANAPE; Fig. 3a); and by their cluster in the turnover analysis (Fig. 3c and e). (TIF 15703 kb) [file 12915_2017_435_MOESM10_ESM.tif]

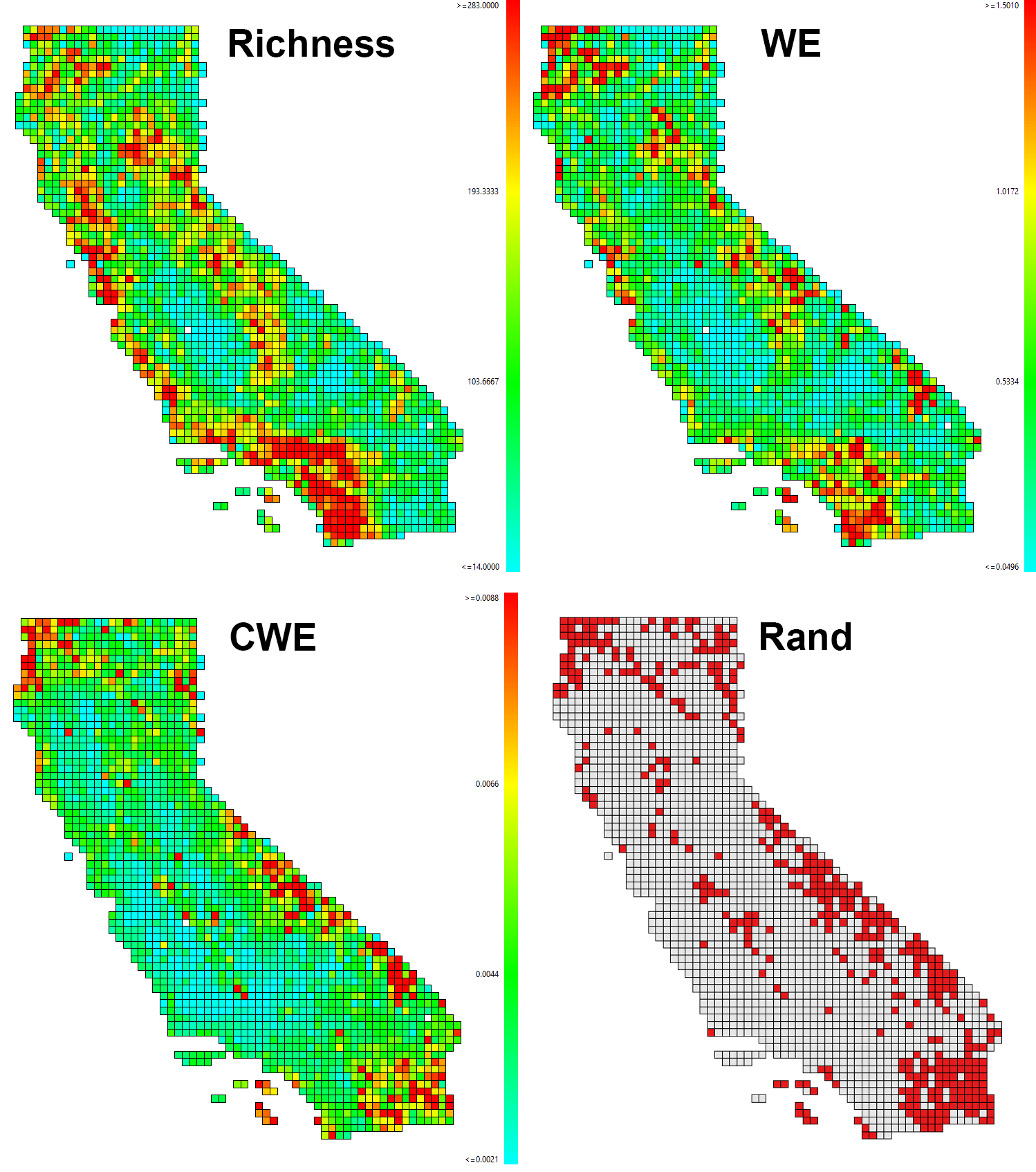

Supplement: Supplementary file 11 — Diversity analyses measured using terminal clades (OTUs) as taxa (i.e., without using a phylogeny). Clockwise from left, Richness, weighted endemism (WE), controlled weighted endemism (CWE), and significantly high endemism following randomization (Rand). (TIF 1622 kb) [file 12915_2017_435_MOESM11_ESM.tif]
